# Most neutralizing human monoclonal antibodies target novel epitopes requiring both Lassa virus glycoprotein subunits

**DOI:** 10.1038/ncomms11544

**Published:** 2016-05-10

**Authors:** James E. Robinson, Kathryn M. Hastie, Robert W. Cross, Rachael E. Yenni, Deborah H. Elliott, Julie A. Rouelle, Chandrika B. Kannadka, Ashley A. Smira, Courtney E. Garry, Benjamin T. Bradley, Haini Yu, Jeffrey G. Shaffer, Matt L. Boisen, Jessica N. Hartnett, Michelle A. Zandonatti, Megan M. Rowland, Megan L. Heinrich, Luis Martínez-Sobrido, Benson Cheng, Juan C. de la Torre, Kristian G. Andersen, Augustine Goba, Mambu Momoh, Mohamed Fullah, Michael Gbakie, Lansana Kanneh, Veronica J. Koroma, Richard Fonnie, Simbirie C. Jalloh, Brima Kargbo, Mohamed A. Vandi, Momoh Gbetuwa, Odia Ikponmwosa, Danny A. Asogun, Peter O. Okokhere, Onikepe A. Follarin, John S. Schieffelin, Kelly R. Pitts, Joan B. Geisbert, Peter C. Kulakoski, Russell B. Wilson, Christian T. Happi, Pardis C. Sabeti, Sahr M. Gevao, S. Humarr Khan, Donald S. Grant, Thomas W. Geisbert, Erica Ollmann Saphire, Luis M. Branco, Robert F. Garry

**Affiliations:** 1 Section of Infectious Disease, Department of Pediatrics, Tulane University School of Medicine, 1430 Tulane Avenue, New Orleans, Louisiana 70112, USA; 2 Department of Immunology and Microbial Science, Scripps Research Institute, 10550 North Torrey Pines Road, La Jolla, California 92037, USA; 3 Department of Microbiology and Immunology, University of Texas Medical Branch at Galveston, 301 University Boulevard, Galveston, Texas 77555, USA; 4 Department of Microbiology and Immunology, Tulane University School of Medicine, 1430 Tulane Avenue, New Orleans, Louisiana 70112, USA; 5 Autoimmune Technologies, LLC, 1010 Common St #1705, New Orleans, Louisiana 70112, USA; 6 Department of Biostatistics and Bioinformatics, Tulane School of Public Health and Tropical Medicine, New Orleans, Louisiana 70112, USA; 7 Corgenix, Inc., 11575 Main Street #400, Broomfield, Colorado 80020, USA; 8 Zalgen Labs, LLC, 20271 Goldenrod Lane, Suite 2083, Germantown, Maryland 20876, USA; 9 Department of Microbiology and Immunology, University of Rochester, 601 Elmwood Avenue, Rochester, New York 14642, USA; 10 Infectious Disease Genomics, Scripps Translational Science Institute, 10550 N Torrey Pines Road, La Jolla, California 92037, USA; 11 Viral Hemorrhagic Fever Program, Kenema Government Hospital, 1 Combema Road, Kenema, Sierra Leone; 12 Department of Laboratory Sciences Polytechnic College, 2 Combema Road, Kenema, Sierra Leone; 13 Ministry of Health and Sanitation, 4th Floor Youyi Building, Freetown, Sierra Leone; 14 Department of Medicine, Institute of Lassa Fever Research and Control, Irrua Specialist Teaching Hospital, Km. 87, Benin/Auchi Road, Irrua, Nigeria; 15 Department of Biological Sciences, College of Natural Sciences, Redeemer's University, Off Gbongan–Oshogbo Road, Ede, Nigeria; 16 African Center of Excellence for Genomics of Infectious Disease (ACEGID), Redeemer's University, Off Gbongan–Oshogbo Road, Ede, Nigeria; 17 Section of Infectious Disease, Department of Internal Medicine, Tulane University School of Medicine, Tulane University School of Medicine, 1430 Tulane Avenue, New Orleans, Louisiana 70112, USA; 18 Department of Organismic and Evolutionary Biology, Center for Systems Biology, Harvard University, 1350 Massachusetts Avenue, Cambridge, Massachusetts 02138, USA; 19 Center for Systems Biology, Broad Institute of Massachusetts Institute of Technology (MIT) and Harvard, 415 Main Street, Cambridge, Massachusetts 02142, USA; 20 Department of Immunology and Infectious Disease, Harvard School of Public Health, 677 Huntington Avenue, Boston, Massachusetts 02115, USA; 21 Department of Medicine, University of Sierra Leone, Freetown, Sierra Leone; 22 The Skaggs Institute for Chemical Biology, The Scripps Research Institute, 10550 North Torrey Pines Road, La Jolla, California 92037, USA

## Abstract

Lassa fever is a severe multisystem disease that often has haemorrhagic manifestations. The epitopes of the Lassa virus (LASV) surface glycoproteins recognized by naturally infected human hosts have not been identified or characterized. Here we have cloned 113 human monoclonal antibodies (mAbs) specific for LASV glycoproteins from memory B cells of Lassa fever survivors from West Africa. One-half bind the GP2 fusion subunit, one-fourth recognize the GP1 receptor-binding subunit and the remaining fourth are specific for the assembled glycoprotein complex, requiring both GP1 and GP2 subunits for recognition. Notably, of the 16 mAbs that neutralize LASV, 13 require the assembled glycoprotein complex for binding, while the remaining 3 require GP1 only. Compared with non-neutralizing mAbs, neutralizing mAbs have higher binding affinities and greater divergence from germline progenitors. Some mAbs potently neutralize all four LASV lineages. These insights from LASV human mAb characterization will guide strategies for immunotherapeutic development and vaccine design.

## Abstract (web summary)

Lassa virus can cause haemorrhagic fever for which no specific treatment currently exists. Here the authors have cloned 113 monoclonal antibodies from the survivors of Lassa infection and show that the majority of neutralizing antibodies target a complex of GP1 and GP2 viral proteins.

## Graphical abstract

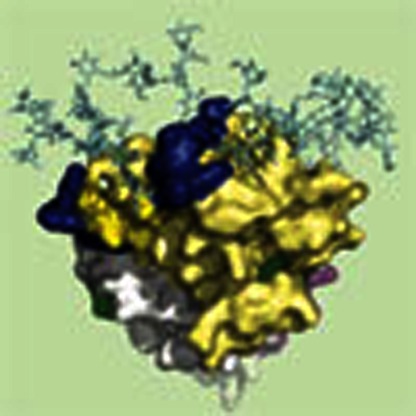

Infection with Lassa virus (LASV), a member of the *Arenaviridae*, results in a spectrum of illness from inapparent infection to Lassa fever, a severe multisystem disease that often has haemorrhagic manifestations. LASV infection is acquired primarily via exposure to the urine and faeces of its reservoir *Mastomys natalensis*, the natal mastomys or multimammate ‘rat' (ref. 1). Tens of thousands of Lassa fever cases are estimated to occur annually in West Africa, with Sierra Leone, Guinea, Liberia and Nigeria reporting the highest incidences^2^. There are three diverse LASV lineages in Nigeria (I–III), but a single lineage (IV) in Sierra Leone^3^,^4^. In Sierra Leone the case fatality rate of patients presenting while viraemic is 69% (ref. 5).

The lack of an approved therapeutic or vaccine, potential for geographic expansion of the rodent reservoir, ease of procurement and weaponization of the virus, and the possible emergence of new viral strains support recommendations for enhanced surveillance and preparedness for Lassa fever^6^. Human monoclonal antibodies (mAbs) can be exploited to gain valuable insights into immune responses to pathogens. Determining the sites at which mAbs bind to pathogen proteins identifies epitopes recognized by the humoral immune system, and may reveal mechanisms of immune surveillance, evasion or escape^7^,^8^,^9^. Structural and biophysical comparisons of protective antibodies to their germline precursors traces their ontogeny, which can guide vaccine development^10^. Human mAbs also have potential for use as immunotherapeutics, with fewer developmental steps and regulatory hurdles than antibodies from other species that require humanization and may elicit immune reactions^11^.

Arenaviruses encode a polyprotein referred to as the glycoprotein complex (GPC), which is processed into GP1 and GP2 and an unusual stable signal peptide (SSP)^12^. GP1 is responsible for receptor binding, while GP2 mediates fusion of the virion with a cell membrane. SSP is required for proper processing of GPC and is retained as part of the complex^13^. To date the epitopes of LASV glycoproteins that elicit protective antibody responses in naturally infected human hosts have not been identified or characterized. To elucidate the antigenic structure of LASV glycoproteins, we isolated and characterized mAbs derived from West African patients who had survived Lassa fever and who had developed sustained antibody titres to LASV antigens months or years into convalescence. The dominant class of neutralizing human mAbs target novel epitopes requiring both Lassa virus glycoprotein subunits. The elucidation of the antigenic structure of the surface glycoproteins of LASV can guide future efforts to derive immunotherapeutics and vaccines.

## Results

### Generation of mAbs

We generated 113 molecularly cloned human mAbs that recognize LASV GPC. These mAbs were derived from B cells of 17 subjects with previous LASV infection (Supplementary Table 1). Fifteen subjects were from Sierra Leone and two were from Nigeria. All but one of the mAbs were first detected by enzyme-linked immunosorbent assay (ELISA) reactivity with an unprocessed insect cell-produced recombinant GPC (Josiah strain lineage IV) lacking the transmembrane and intracytoplasmic domains (rGPe) as the capture antigen. The exception was 8.9F, which did not bind to rGPe in ELISA, but was detected by its ability to neutralize a pseudovirus expressing LASV GPC.

**Figure 1:**
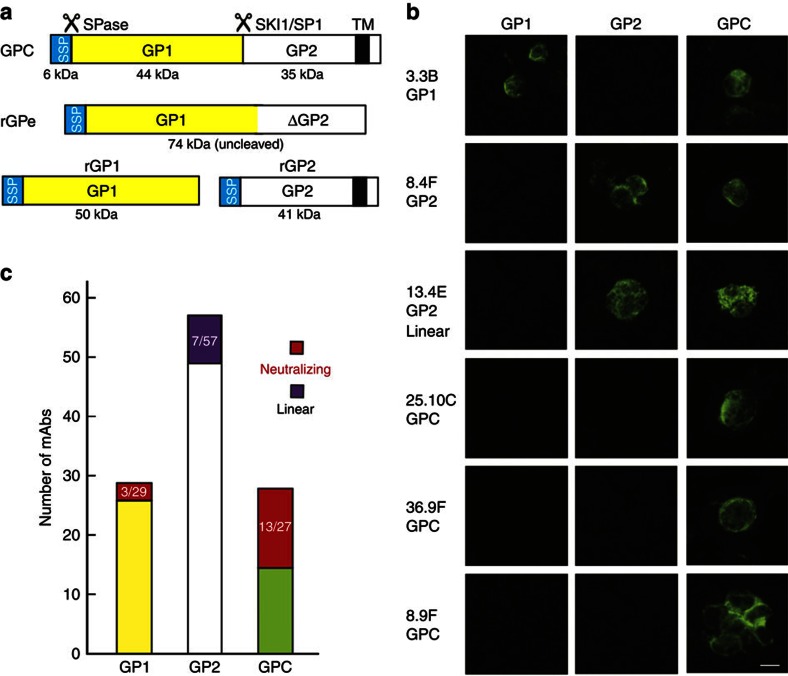
Subunit specificity of LASV mAbs. (**a**) The LASV GPC is synthesized as a precursor protein. Signal peptidase (SPase) cleaves the small SSP, which remains associated with the complex. SKI protease (SK1/S1P) cleaves GPC into GP1 and GP1. A recombinant LASV (Josiah strain, lineage IV) GPC ectodomain construct (rGPe) was expressed that substituted an uncleavable linker for the SK1 site and deleted the transmembrane and extracellular domains. Constructs expressing recombinant SSP-GP1 (rGP1) and SSP linked to GP2 were also utilized. Molecular weights are indicated in kiloDaltons (kDa). (**b**) Examples of immunofluorescence assays with unfixed, air-dried cells expressing GP1, GP2 or GPC. All assays were performed two or more times. Scale bar, 10 μm. (**c**) Distribution of 113 mAbs by subunit specificity, neutralizing activity and reactivity to linear peptides.

**Figure 2:**
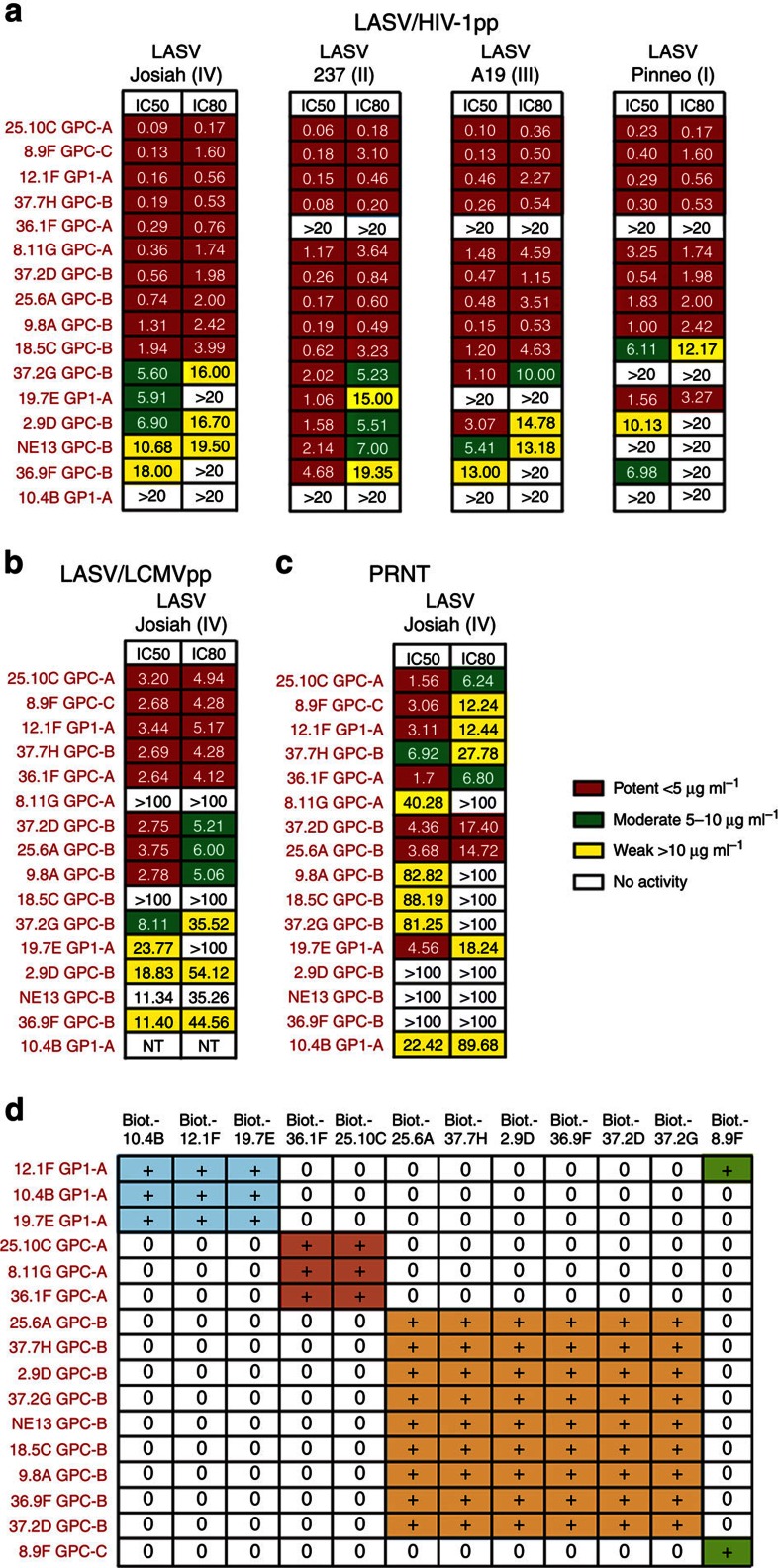
LASV neutralization by mAbs and assignment of competition groups. Neutralizing activity of mAbs was evaluated in two pseudovirus assays and by a PRNT. (**a**) LASVpp expressing GPC representing the four LASV lineages (I–IV) and the HIV-1 core. (**b**) LASVpp expressing GPC representing LASV lineage IV and the LCMV core. (**c**) PRNT. The inset indicates the potency (heat map) for IC_50_ and IC_80_ neutralization by each of the mAbs tested in **a**–**c**. All three pairwise correlation coefficients were significantly different at the 5% significance level according to both Pearson's and Spearman's approaches. (**d**) Cross-competition analysis was performed pairwise with biotinylated (Biot.) mAbs. The analysis places neutralizing mAbs into four competition groups. 8.9F competes with itself and its binding is blocked by 12.1F. Results in Fig. 2 are representative of at least two experiments performed for each assay.

LASV GPC consists of a SSP and GP1 and GP2 subunits (Fig. 1a and Supplementary Figs 1 and 2). To define subunit specificity of the mAbs, we used an immunofluorescence assay to test mAb reactivity with air-dried, unfixed smears of HEK293T cells expressing either rGP1 or rGP2 alone, or full-length GPC (Fig. 1b). This assay detected all mAbs, including 8.9F. No mAb was reactive with untransfected cells. Twenty-nine mAbs were reactive with GP1, including three neutralizing mAbs (Figs 1c and 2). Fifty-seven mAbs recognized GP2, but none of these exhibited neutralizing activity (Fig. 1c). Seven mAbs reacted with peptides representing three linear epitopes in GP2 (Supplementary Fig. 3); the remaining mAbs likely recognize conformational epitopes. Twenty-seven mAbs reacted with cells expressing full-length GPC, but did not react with either rGP1 or rGP2 expressed individually. Remarkably, thirteen of these anti-GPC mAbs were neutralizing. This GPC-reactive set constitutes the major group of neutralizing mAbs identified.

### Neutralization properties of mAbs

Neutralizing activities of mAbs were evaluated in two pseudovirus assays and in a standard plaque reduction neutralization test (PRNT) with authentic LASV. In all, 15 of the 113 mAbs neutralized LASV pseudoparticles (LASVpp) expressing GPC of the lineage IV Josiah strain (Fig. 2a and Supplementary Fig. 4). These mAbs were tested in the same assay against LASVpp containing GPC of the three other LASV lineages. A heat map of the IC_50_ and IC_80_ values shows that those with the greatest potency and breadth against all four LASV lineages were 25.10C, 12.1F, 8.9F, 37.2D, 37.7H, 25.6A and 9.8A (Fig. 2a). The remaining mAbs showed weaker and variable potency. 37.2G, 2.9D and NE13 had little or no activity against the Pinneo strain (lineage I), 19.7E failed to neutralize the A19 strain (lineage III) and 36.1F neutralized only the Josiah strain (lineage IV).

To confirm these findings, we tested a subset of mAbs for activity against LASV lineage IV Josiah using an lymphocytic choriomeningitis virus (LCMV)-based pseudovirus assay and a standard PRNT with authentic LASV. The six antibodies that were the most potent against human immunodeficiency virus (HIV)-1 pseudoviruses (25.10C, 8,9F, 12.1F, 36.1F, 37.2D and 25.6A) were also the most potent against LCMV-based LASV pseudoparticles and authentic LASV although IC_50_ and IC_80_ values were somewhat higher in LCMV-based pseudoparticle and PRNT assays (Fig. 2c). Further, several mAbs that neutralized LASVpp either did not neutralize authentic LASV (36.9F, NE13 and 2.9D) or showed weak activity (8.11G, 37.2G, 9.8A and 18.5C). A significant difference was that 10.4B showed intermediate neutralizing activity in the PRNT, but did not neutralize in either LASVpp system.

### Cross-competition groups

Using an ELISA with ConA-captured LASV GPC from Triton X-solubilized cells as antigen, we performed cross-competition assays with the 16 LASV-neutralizing mAbs to determine whether they recognized independent binding sites. We identified four competition groups of neutralizing mAbs (Fig. 2d). One is comprised of the GP1 mAbs 12.1F, 19.7E and 10.4B. The other three contain the anti-GPC mAbs. GPC-A contains 25.10C, 36.1F and 8.11G; GPC-B includes 37.2D, 37.7H, 37.2G, 36.9F, 25.6A, 2.9D, 18.5C and NE13; and GPC-C contains one mAb, 8.9F, which competes only with itself. Interestingly, the anti-GP1 mAb 12.1F partially blocks 8.9F and 8.11G binding, but 8.9F and 8.11G do not block binding of 12.1F. Several non-neutralizing mAbs (20.5G and 38.8B) cross-compete with GPC-B mAbs and are included in this group (Supplementary Table 1). Non-neutralizing mAbs that react with GP1 expressed alone fail to cross-compete with GP1-A mAbs, but variably cross-compete with each other and recognize a cluster of epitopes tentatively designated GP1-B. There are at least two additional groups of non-neutralizing mAbs, GP2-A and GP2-B, that recognize conformational epitopes on GP2. Three additional groups of non-neutralizing GP2 mAbs that recognize linear peptides are designed GP2-L1, -L2 and -L3.

### Immunoprecipitation of GP1 and GP2 by neutralizing mAbs

**Figure 3:**
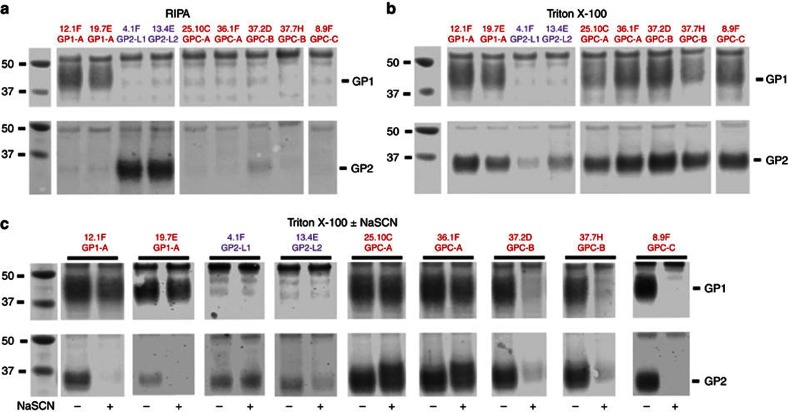
Immunoprecipitation with anti-GPC mAbs in RIPA buffer, Triton X-100 and Triton X-100±NaSCN. LASV mAbs indicated were used as capture antibodies in immunoprecipitation studies employing lysates from cells expressing LASV GPs (GPC, rGP1 and rGP2). Immunoprecipitated proteins were resolved by SDS–PAGE, transferred to nitrocellulose. Western blots were probed with mouse mAbs to either GP1 (upper gel image in each panel) or GP2 (lower image). (**a**) Immunoprecipitation with the indicated mAbs was performed with cell lysates prepared in RIPA buffer. (**b**) Immunoprecipitation was performed with cell lysates prepared in Triton X-100-containing buffer. (**c**) mAb-GPC immune complexes bound to magnetic protein A beads were treated or not treated with NaSCN before SDS–PAGE and western blotting. Gel lanes have been reordered for clarity. Uncropped western blot images are presented in Supplementary Fig. 6. Experiments were repeated at least twice.

To characterize the interactions of mAbs with LASV GPC and its subunits, we performed immunoprecipitation assays with lysates of GPC-expressing cells prepared either in radioimmunoprecipitation assay (RIPA) buffer or in buffer containing 1% Triton X (Fig. 3 and Supplementary Fig. 5). Representative GP1 mAbs (12.1F and 19.7E) or GP2 mAbs (4.1F and 13.4E) precipitated only GP1 or GP2, respectively, from RIPA buffer lysates, whereas the anti-GPC mAbs were non-reactive (Fig. 3a). By contrast, anti-GPC mAbs invariably co-precipitated GP1 and GP2 equally well from Triton X lysates (Fig. 3b). However, under these conditions 19.7E and 12.1F also co-precipitated both subunits, despite their known GP1 specificity. GP2 mAbs 4.1F and 13.4E were less reactive with GP2 in Triton X lysates than in RIPA lysates and showed minimal or no co-precipitation. These results suggested that processed GP1 and GP2 form stable complexes within transfected cells and that the stronger ionic and anionic detergent mixture in RIPA buffer disrupts GP1–GP2 interactions that are critical for recognition by anti-GPC mAbs. Dissociation of GP1 and GP2 by RIPA buffer permits greater reactivity with the GP2 mAbs 4.1F and 13.4E.

To determine if anti-GPC mAbs could bind to one subunit more strongly than the other, we performed immunoprecipitation assays in which mAb–GP1–GP2 complexes formed with Triton X lysates were exposed to the chaotropic agent sodium thiocyanate (NaSCN) at 1.5 M (Fig. 3c). As noted above, the GP1 mAbs 12.1F and 19.7E both co-precipitated GP1 and GP2. NaSCN did not affect precipitation of GP1 by either mAb, but eliminated GP2 reactivity, thus uncovering the preferred mAb specificity.

For the anti-GPC mAbs, two patterns were observed. In the first pattern, shown with 25.10C and 36.1F, NaSCN treatment did not affect mAb binding to either subunit; band densities for GP1 and GP2 were equivalent in these on western blots. The same pattern was observed with mAbs 36.9F, 36.7G and 18.5C (Supplementary Figs 5 and 6). These mAbs likely bind epitopes that bridge both subunits in a stable complex. In the second pattern, exemplified by 37.2D, 37.7H and 8.9F, NaSCN treatment caused almost complete and equal loss of both GP1 and GP2 reactivity (Fig. 3c). mAbs 9.8A, 8.11G, 25.6A, NE13 and 2.9D showed similar patterns (Supplementary Figs 5 and 6). These mAbs may also bind epitopes that bridge GP1 and GP2, or that require the complex of the two to maintain the structural integrity of the epitope. Importantly, NaSCN treatment of immune complexes did not uncover preferential binding to either GP1 or GP2 by these anti-GPC mAbs.

### Cross-reactivities between Old and New World arenaviruses

**Figure 4:**
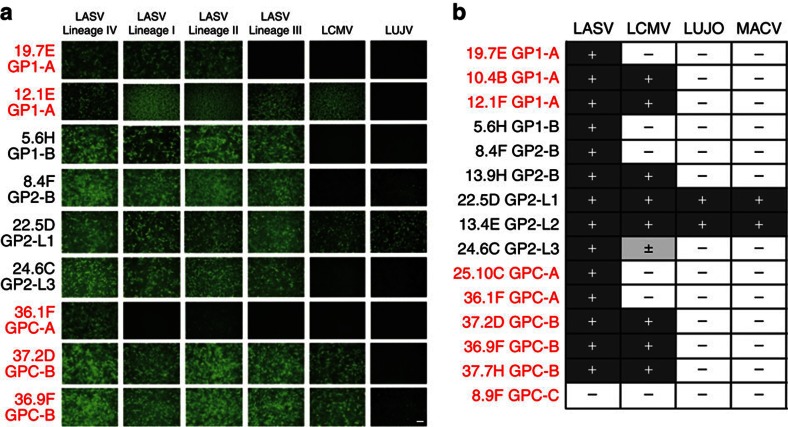
Cross-reactivity of mAbs isolated from survivors of LASV lineage IV infection with glycoproteins of other arenaviruses. Cross-reactivity of human mAbs produced after natural infection with LASV lineage IV was investigated by immunofluorescence or by antibody-capture ELISA. (**a**) Eukaryotic expression vectors encoding GPCs of various OW arenaviruses, including LASV lineages I–IV, LCMV and LUJV were transfected into HEK293T cells. Fixed cell monolayers were then incubated with LASV mAbs and mAb binding was detected by indirect immunofluorescence. An expanded panel of mAbs and OW arenavirus GPCs is presented in Supplementary Fig. 7. Scale bar, 10 μm. (**b**) GPC from the OW arenaviruses LASV (Josiah strain, lineage IV), LCMV, LUJV and the New World arenavirus MACV were expressed and purified. Reactivity of the indicated mAbs was assessed by ELISA. An expanded panel of mAbs is presented in Supplementary Fig. 8a. Data are pooled from at least four independent experiments.

Cross-reactivity of a subset of mAbs was determined by immunofluorescence assay with cells expressing GPC of the four lineages of LASV or other representative Old World (OW) arenaviruses (Fig. 4a and Supplementary Fig. 7). mAb 19.7E (and 10.4B) bound LASV GPC from lineages I, II and IV, but failed to bind GPC from lineage III. In agreement with its neutralizing activity, GPC-A antibody 36.1F bound only to LASV lineage IV, the lineage that circulates in Sierra Leone, whereas GPC-B mAbs bound effectively to GPC from each lineage. mAbs 12.1F, 37.2D, 36.9F, 37.2G, 18,5C and 9.8A bound to GPC of LCMV, but not to Lujo virus (LUJV) or other divergent OW arenaviruses. GP2-L1 antibodies 25.6E, 22.5D and 8.8B showed broad cross-reactivity with OW arenaviruses, but GP2-L3 mAb 24.6C only reacted to LASV. 8.9F was non-reactive with LASV GPC in fixed transfected cells.

In testing by ELISA, we found that the GP1-A antibodies 10.4B and 12.1F, as well as each of the GPC-B mAbs tested cross-react with LCMV GPC (Fig. 4b and Supplementary Fig. 8a). However, another GP1-A mAb (19.7E), GPC-A antibodies and most GP1-B antibodies did not cross-react with LCMV GPC. About half of the GP2-B antibodies tested cross-reacted with LCMV GPC. Most mAbs recognizing conformational epitopes (GP1 and the GPC mAbs), except a subset of GP2-B mAbs, also did not cross-react with GPC of LUJV, a divergent OW arenavirus, or with GPC of MACV, a New World arenavirus. In contrast, GP2-L1 and -L2 mAbs that recognize linear peptides demonstrated broad cross-reactivity, binding GPC of LCMV, LUJV and MACV. The GP2-L3 mAb 24.6C showed weak cross-reactivity with LCMV GPC. Overall, mAb cross-reactivities correlated with the degree of conservation of the putative epitopes (Supplementary Fig. 2).

### Epitope mapping using deletion constructs

**Figure 5:**
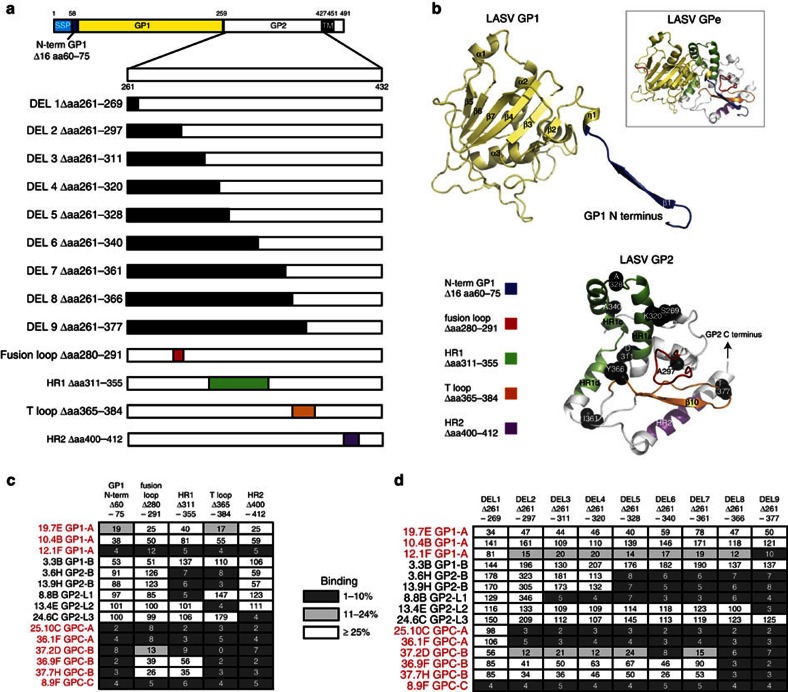
Mapping of putative epitopes recognized by LASV mAbs by deletion mutagenensis. Wild-type recombinant LASV GPC was engineered with deletions of amino-acid sequences to map putative B-cell epitopes on LASV glycoproteins. (**a**) Location of the N-terminal (N-term) deletion of GP1, nested deletions in GP2 and mutations that delete the fusion loop, HR1, T-loop and HR2 are indicated. Alpha helices and beta sheets are labelled according to Hastie *et al*.^14^. (**b**) Location of deletions in engineered GPC are mapped to structural models of GP1 and GP2 derived from threading of the LASV GPC ectodomain to the structure of LCMV GPC. Deletions are colour coded as in **a**. The location of the last amino acid of each of the nested deletions in GP is shown as a black sphere. The inset depicts the LASV GPe structural model and interaction of the N terminus of GP1 with the fusion loop and T-loop of GP2 (see also Supplementary Fig. 2a). (**c**) ELISA reactivity of selected mAbs to GPC constructs containing deletions of the N terminus of GP1 and indicated regions of GP2. ELISA was performed with GPC in Triton X lysates captured in wells coated with ConA. We normalized the binding of mAbs to each mutant by comparing optical density values observed with each mAb reacted with mutated GPC to that observed with unmutated GPC. Values indicate per cent binding with mutant compared with unmutated control. Mutations that resulted in binding that was <10% of binding to unmutated GPC were considered as having a significant effect on mAb binding. Mutations that reduced binding to 10% or less of control or 11–24% of control are highlighted. Reactivity of GP1-B and GP2-L1, L2 and L3 mAbs with each mutant reflected the degree to which mutations had global effects on glycoprotein conformation. An expanded panel of mAbs is presented in Supplementary Fig. 7b. (**d**) ELISA reactivity of selected mAbs to GPC constructs containing increasingly larger deletions in GP2. An expanded panel of mAbs is presented in Supplementary Fig. 8c. The results shown are representative of multiple (*n*>4) independent experiments.

To map regions or sites in GP1, GP2 and GPC that are critical for mAb binding, we tested a panel of mAbs for reactivity with a series of deletion mutants in full-length GPC (Fig. 5a). Given that LASV and LCMV share 63% sequence identity and several of the conformationally sensitive GPC-specific antibodies cross-react with LCMV GPC, the recently solved structure of the ectodomain of LCMV GPC (rGPe)^14^ was used to model the LASV GPe protomer and to depict the location of the deletion mutations (Fig. 5b and Supplementary Fig. 1). In the prefusion LCMV GPe structure, the N terminus of GP1 makes extensive interactions with GP2, notably with the fusion loop, the T-loop, and the two heptad repeat regions (HR1 and HR2). Deletion of the first 16 amino acids of LASV GP1 (Δaa60–75) disrupted binding by 12.1F, as well as all anti-GPC-neutralizing mAbs, while having only minimal to modest effects on remaining mAbs tested (Fig. 5c and Supplementary Figs 1b and 8b).

LASV GP2 is a class I viral fusion protein (VFP) that forms two heptad repeats in the post-fusion configuration^15^. The sequences involved in virus:cell membrane fusion at the N terminus of GP2 (aa261–297) include a peptide (aa261–279) and a disulfide bonded loop (C279–C292). Deletion of a portion of the fusion loop (Δaa280–291) resulted in complete loss of binding by the neutralizing mAbs 8.9F, 25.10C, 8.11G and 36.1F, and diminished binding by the neutralizing mAbs 12.1F, 37.2D and 9.8A. In the prefusion configuration of LCMV-BGP2 heptad repeat 1 (HR1) is separated into segments that interact with GP1 and probably contribute to GPC stability^14^. Deletion of HR1 (Δaa311–355) abolished binding by non-neutralizing GP2 mAbs (GP2-B) and GP2-L1 mAbs. HR1 deletion also disrupted binding by the same mAbs that were affected by the fusion loop deletions, suggesting that binding of these antibodies is dependent on maintenance of the prefusion conformation of GP2. The T-loop of GP2, between the two heptad repeats, interacts with both the N terminus of GP1 and the fusion loop. Deletion of the T-loop (Δaa365–384) profoundly disrupted reactivity of most neutralizing GP2-B mAbs. In addition, the T-loop deletion abolished binding by the non-neutralizing HR1-dependent GP2-B mAbs, as well as 13.4E and 1.1B that recognize a linear epitope within the T-loop. Deletion of the part of HR2 (Δaa400–412) that interacts with the N terminus of GP1 abolished binding of all neutralizing mAbs except 19.7E and 10.4B, and also affected binding of the non-neutralizing anti-GPC-B mAb 20.5G and the non-neutralizing anti-HR2 linear epitope recognized by 24.6C GP2-L3, which binds a peptide epitope aa400–412.

To corroborate these results we engineered a series of GPC constructs containing increasingly larger deletions in GP2. Deletion of the N-terminal portion of the fusion loop (DEL1, Δaa261–269) affected only 8.9F binding (Fig. 5d). Deletion of the entire fusion loop (DEL2; Δaa261–297) had the same effect as the partial fusion loop deletion discussed above. DEL3 (Δaa261–311) removed the linear epitope recognized by mAb 4.1F and other antibodies in group GP2-L1. Binding of one mAb 7.1B (GP1-A) was abrogated by DEL 4 (Δaa261–320), which removes the first segment of HR1 (Supplementary Table 1). In contrast, GP2-B mAbs bound effectively to DEL4, but did not bind to DEL5, which also deletes half of HR1 (Δaa261–328), again demonstrating that binding of GP2-B mAbs depends on the integrity HR1 region (Fig. 5d). Remarkably, GPC-B mAbs tolerated GP2 deletions of up to 100 amino acids (DEL6 Δaa261–340 and DEL7 Δaa261–361) and their binding was not disrupted until the DEL8 (Δaa261–366) mutant removed the first few amino acids in the T-loop formed by disulfide bonds between C364 and C385. This deletion also abolished binding by the non-neutralizing GPC-B mAbs 20.5G and 38.8B that cross-compete with GPC-B-neutralizing mAbs. DEL9 (Δaa261–377) removes a linear epitope within the T-loop, causing loss of binding by GP2-L3 mAbs 13.4E and 1.1B. Non-neutralizing mAbs binding to GP1 (2.4F and 3.3B) and to GP2-L3 (24.6C) were not affected by any of the GP2 deletion mutants.

### Epitope mapping using site-directed mutagenesis

**Figure 6:**
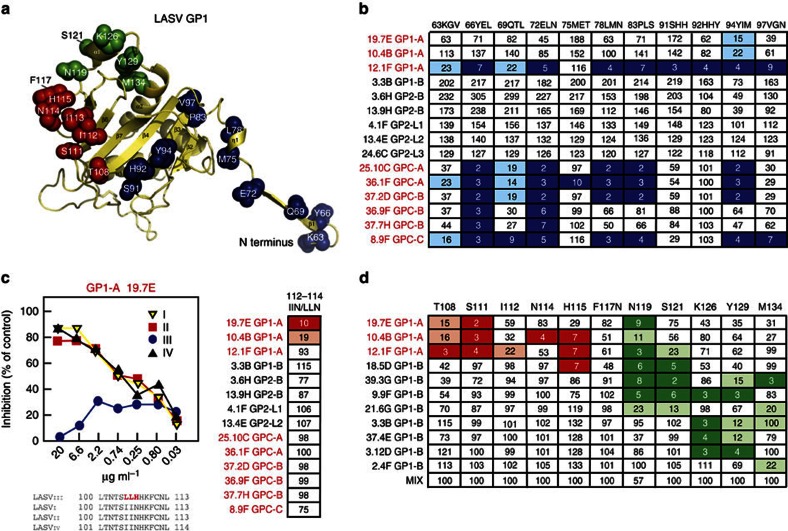
Mapping of putative epitopes recognized by LASV mAbs by site-directed mutagenesis. Wild-type recombinant LASV GPC was engineered with altered amino-acid sequences to map putative B-cell epitopes on LASV glycoproteins. (**a**) Location of mutations in engineered LASV glycoproteins are mapped to a structural model of GP1. (**b**) Binding of mAbs to GPC constructs in which the indicated sets of three amino acids in the amino terminus of GP1 were mutated to three alanines. ConA-capture ELISA performed as in Fig. 5; results expressed as per cent of binding to unmutated control. An expanded panel of mAbs is presented in Supplementary Fig. 8d. (**c**) 19.7E fails to neutralize LASVpp expressing GPC from lineage III LASV. The sequence of lineage IV GP1 was partially altered to the sequence in lineage III GPC by changing the sequence IIN to LLN. Binding of mAbs was quantified by ConA ELISA as described above. Mutations that reduced binding to 10% or less of control or 11–24% of control are highlighted. (**d**) Binding of mAbs to GP1 constructs in which the indicated single amino acids in GP1 were mutated to alanine and expressed as D7-tagged proteins. Binding of mAbs to mutant and control GP1-D7 constructs captured in wells coated with mAb JR-52. A mixture of anti-GP1 mAbs was included as a control. Values indicate per cent binding of mutants compared with unmutated GP1. The results shown are representative of multiple (*n*>4) independent experiments.

We carried out site-directed mutagenesis studies to better define residues in GP1 that affect antibody recognition (Fig. 6a). A series of triple-alanine substitutions in the N terminus of GP1 in full-length GPC between amino acid residues K63 through N98 affected individual mAbs differently (Fig. 6b and Supplementary Fig. 8c). YEL66-68AAA and ELN71-74AAA triple mutations each abolished binding by the same neutralizing mAbs that were affected by deletion of aa60–75 in the N-terminal region, while mutation of residues QTL69-71AAA had less drastic effects. The MET75-77AAA mutation abolished binding by only mAb 36.1F. Effects on mAb binding with AAA mutants at LMN77-79, PLS83-84 and YIM94-96 were similar to those seen with the fusion loop deletion mutant (Δaa280–291); namely loss of binding by 12.1F and GPC-A mAbs (36.1F, 25.10C and 8.11G), with somewhat diminished binding to 37.2D and 9.8A. Mutants SHH91-93AAA and HHY92-94AAA uniquely affected 12.1F binding. Mutant VGN97-99AAA affected both 12.1F and 8.9F binding. The residues aa91–97 are part of a beta sheet (β5) that may be a target for the binding of these antibodies. In addition, histidines at aa92 and 93 have been implicated in LAMP1 receptor binding by GP1 (ref. 16).

mAb19.7E neutralizes LASVpp expressing GPC of lineage IV, but fails to neutralize LASVpp expressing GPC from lineage III (Fig. 6b). Lineage III differs from other lineages by an LLH in place of an IIN at positions 112–114. Altering these residues in GP1 from lineage IV from IIN to LLN reduced the binding of 19.7E and 10.4B without affecting binding by other mAbs. Interestingly, although 12.1F is in the same GP1-A competition group as 19.7E and 10.4B, its binding is not affected by this mutation. We introduced single-alanine substitutions of additional residues in GP1 to further delineate the putative epitopes of the GP1 mAbs (Fig. 6c and Supplementary Fig. 8d). Mutation of residue S111 to alanine reduced the binding of all three GP1-A mAbs 19.7E, 10.4B and 12.1F. These studies also confirmed the importance of residues adjacent to aa112 in binding of GP1-A mAbs and suggested that aa119–129 contribute to the binding of mAb in competition group GP1-B.

### Location of putative epitopes on LASV GPC

**Figure 7:**
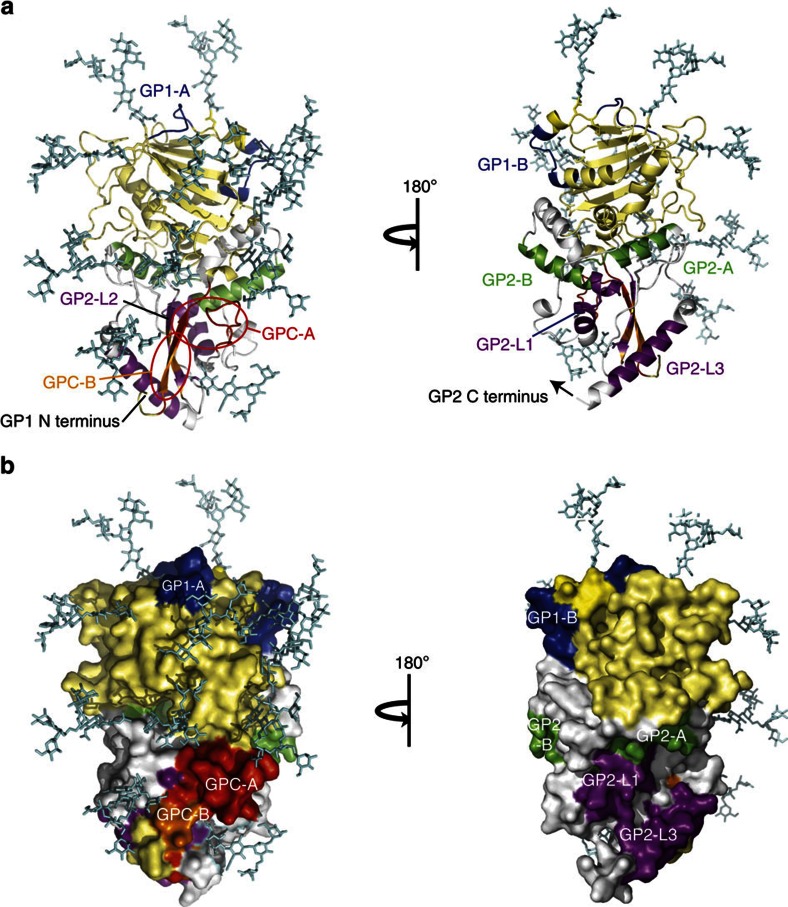
Location of putative epitopes on a structural model of LASV GPC. Antibody epitopes are mapped onto the model of the LASV GP ectodomain monomer. (**a**) Ribbon view. (**b**) Surface view. GP1, outside putative epitopes, is coloured yellow. GP2, outside putative epitopes, is coloured white. Epitope colour code: GP1-A and GP1-B, blue; GP2-A and GP2-B, green; GP2-L1-3, purple; GPC-A, red; and GPC-B, orange. The location of the conformational epitope of GPC-C mAb 8.9F is unknown.

As is the case with the LCMV GPC structure, all potential N-linked glycosylation sites are present on the upper face of the modelled LASV GPC, forming a glycan shield (Fig. 7 and Supplementary Fig. 1c). The putative epitopes recognized by mAbs of groups GP1-A, GPC-A and GPC-B correspond to surface loops between β-sheets or helical domains on the glycosylated side that are not fully occluded by glycosylation. GP1-B, GP2-A, GP2-B and linear epitopes GP2-L1 and L3 appear to be accessible to antibody binding from the opposite face on GPC (180^o^ rotation). The linear epitope at aa361–375 recognized by 13.4E and 1.1B (GP2-L2) and the conformational epitope of GPC-B mAbs are comprised in part of a β-sheet that interacts with the amino terminus of GP1.

### Ontogeny of anti-LASV GP mAbs

**Figure 8:**
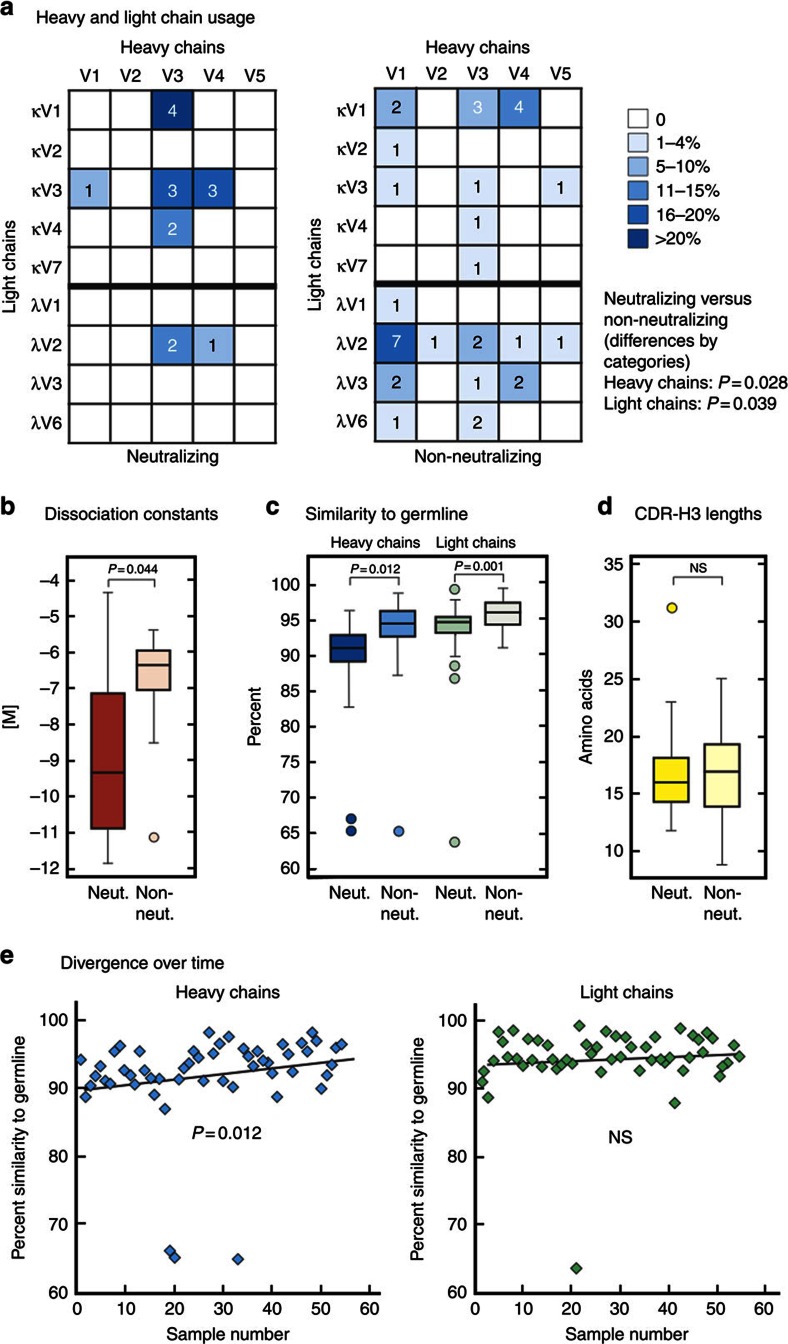
LASV mAbs ontogeny and binding kinetics. Antibody heavy- and light-chain constructs were sequenced and consensus immunoglobulin sequences spanning framework 1 through 4, and CDRs 1–3 were subjected to *in silico* analysis using IMGT/V-QUEST and the abYsis integrated antibody analysis and prediction software. (**a**) Use of heavy and light chains by neutralizing (left) and non-neutralizing mAbs. (**b**) Dissociation constants of neutralizing and non-neutralizing mAbs. (**c**) Divergence from presumed germline genes of heavy and light chains of neutralizing and non-neutralizing mAbs. (**d**) CDR-H3 lengths of neutralizing and non-neutralizing mAbs. (**e**) Similarity to presumed germline genes of heavy and light chains of human mAbs as a function of time between infection and PBMC collection. Neut., neutralizing; Non neut., non-neutralizing.

Sequence analysis of genes encoding the LASV mAbs revealed a significant bias of neutralizing mAbs for use of V3 heavy chains (IGHV3) and lambda light chains (Fig. 8a). Neutralizing antibodies showed a lower dissociation constant (mean of log10 KD±s.d., −8.94±2.62 versus −6.60±1.27; *P*=0.044 by the Wilcoxon Mann–Whitney test, Fig. 8b) suggesting affinity maturation to superior LASV GPC binding. Both the heavy and light chains of non-neutralizing mAbs show moderate divergence from their presumptive germline genes at the nucleotide level, whereas neutralizing mAbs display significantly lower similarities (heavy chains, *P*=0.012; light chains, *P*=0.001) to germline (Fig. 8c). Three LASV mAbs, including two neutralizing mAbs isolated from the same donor, showed pronounced divergence from presumptive germline genes in one or both chains (∼65% identity) (Supplementary Table 1). A correlation between the length of complementarity-determining region (CDR)-H3 domains, which is typically the major antigen-binding domain, and neutralization potential was not observed (Fig. 8d). CDR-H3 lengths of LASV-neutralizing mAbs were variable, ranging from 12 to an unusually long 31 amino acids (Supplementary Table 1). The heavy chains of mAbs, but not the light chains, showed a significant trend (*P*=0.012) to greater divergence from germline with increased time between infection and peripheral blood mononuclear cell (PBMC) collection (Fig. 8e).

## Discussion

We generated and analysed a panel of 113 human mAbs directed against LASV GPC, derived from 17 human survivors of LASV infection in West Africa. The 16 neutralizing mAbs recognize four distinct groups of binding sites. One group contains three mAbs directed against the GP1 subunit. The other three groups each contain mAbs that bind GPC, but not GP1 or GP2 alone. Immunoprecipitation assays performed with and without exposure of mAb-GPC immune complexes to NaSCN strongly suggest that the anti-GPC mAbs recognize complex epitopes that bridge the GP1 and GP2 subunits, or that require the complex of the two to maintain the quaternary nature of the epitope. This class of neutralizing mAbs has not been previously reported for any arenavirus.

Mutagenesis studies guided by a pre-fusion GPC crystal structure for the related OW arenavirus LCMV^14^ defined architectural features of LASV GPC required for binding by the different mAbs. These studies point to critical interactions between GP1 and GP2 in prefusion GPC, the maintenance of which is necessary for binding by most neutralizing mAbs. Notably, with the exception of GP1-specific 19.7E and 10.4B, all neutralizing mAbs are sensitive to deletion or mutagenesis of the N-terminal 16 amino-acid residues of GP1 and deletions of the T-loop and HR2 in GP2. Deletion of the N terminus of GP1 and mutagenesis of critical contacts between GP1 and GP2 likely cause some degree of subunit dissociation that triggers transition to a post-fusion conformation and disrupts exposure of neutralizing mAb-binding sites. Mutagenesis of the fusion loop of GP2 further subdivides GPC-neutralizing antibodies that require both GP1 and GP2 for binding. Those mAbs in group GPC-A are affected by fusion loop deletions or mutations, while those in group GPC-B are significantly less affected. Group GPC-C contains a single mAb 8.9F with a long CDR-H3 region (31 aa). This mAb recognizes a complex quaternary epitope that is affected by an unusually wide variety of mutations in both GP1 and GP2.

The glycan coat of GPC has recently been implicated in shielding LASV against the neutralizing effect of host antibodies^17^. A model of LASV GPC derived by threading the structure of LCMV GPC^14^ suggests that two putative LASV GP1 epitopes map to exposed loops between glycosylation sites on this subunit. GP1-A mAbs that interact with one of these loops are capable of neutralizing LASV by a yet to be determined mechanism. The intimate interactions between the N-terminal extension of GP1 and the fusion and T-loops of GP2 observed in LCMV GPC^14^, are likely to occur in GPC of LASV and other arenaviruses. After exposure to low pH in the endosome arenavirus GPC, like other VFPs, undergoes extensive structural rearrangements, which trigger fusion of the viral and cell membranes. Gaps in the glycan shield of GPC may permit neutralizing antibodies to interact simultaneously with the N-terminal extension of GP1 and either the fusion loop or T-loop of GP2, resulting in blockade of structural alterations required for fusion. Precedence for such a mechanism by neutralizing antibodies that bridge the two subunits of other viral fusion proteins has been established^8^,^18^.

Although mAbs provide potent tools for epitope identification and characterization, relatively few reports have described murine mAbs that react with glycoproteins of the OW arenaviruses LCMV^19^,^20^,^21^ or LASV^22^. LCMV-neutralizing mAbs recognize an epitope designated GP1-A^20^,^23^,^24^, which based on sequences of neutralization escape mutants involves asparagine119 (ref. 25). It is likely that this epitope is similar to that recognized by GP1-A LASV mAbs. Two non-neutralizing LCMV mAbs were mapped to the same cross-reactive linear epitope in the T-loop of LCMV GP2 that is recognized by the GP2-L2 mAbs we describe here^20^. A panel of murine mAbs against LASV glycoproteins was reported^22^. Although a few were neutralizing, they were not further characterized. Studies of mAbs to New World arenaviruses have also been limited, but include mAb directed against the fusion peptide of Junin virus (JUNV) envelope glycoprotein GPC that inhibits pH-induced membrane fusion^26^. Recently, Mahmutovic *et al*.^27^ determined the structure of a neutralizing mouse mAb in complex with JUNV rGP1. The footprint of this mAb corresponds to the putative epitope recognized by neutralizing GP1-A mAbs.

There is no approved Lassa fever vaccine, and the only available treatment, ribavirin, is most effective only during early infection^28^. Passive transfer of survivor plasma is used as a treatment for infection by JUNV, the causative agent of Argentine haemorrhagic fever^29^. Attempts to use convalescent plasma against Lassa virus have met with variable results^30^. Here we demonstrate that the neutralizing humoral response matures over time in convalescent Lassa fever (LF) patients. In addition to our observation that the most effective neutralizing antibodies are directed against particular quaternary assemblies on prefusion GPC, we also find that neutralizing antibodies to LASV have a higher divergence from germline genes and higher binding affinities than non-neutralizing mAbs. Further, these studies provide the initial foundation for understanding the molecular mechanisms of antibody development, antibody binding and antibody-mediated neutralization of LASV in humans. These results may facilitate development of epitope-targeted vaccines or broadly reactive antibody-based therapeutics, both urgently needed in a region of the world now confronted with two co-circulating, highly pathogenic haemorrhagic fever viruses.

## Methods

### Ethics statement

The Tulane University Institutional Review Board, the Sierra Leone Ethics and Scientific Review Committee and the Irrua Specialist Teaching Hospital (ISTH) Ethics Committee approved this project. Patients were referred to the Kenema Government Hospital (KGH) or ISTH Lassa Wards from regional health centres or the hospitals' general wards on the basis of suspicion of Lassa fever. Patients were cared for by trained staffs. All adult subjects provided written informed consent to analyse and publish laboratory and clinical data. A parent or guardian of any child participant provided written informed consent on their behalf.

### Patient samples

Anticoagulated whole blood was obtained from healthy LASV-seropositive adult subjects in Sierra Leone or Nigeria. PBMCs were separated from blood on Ficoll-Hypaque and cells were cryopreserved in liquid nitrogen at the KGH or ISTH. Samples were then transported to Tulane University Health Sciences Center in liquid nitrogen dry shippers and ultimately stored in the vapour phase of liquid nitrogen freezers.

### Cells

The following cell lines were obtained from American Type Culture Collection (ATCC): human embryonic kidney 293T cells (ATCC CRL-11268); African green monkey kidney epithelial Vero cells (ATCC CCL-81); and baby hamster kidney BHK-21 (ATCC CCL-10). These cells were grown in Dulbecco's modified Eagle's medium supplemented with 10% fetal bovine serum (FBS), 2 mM L-glutamine, penicillin (100 U ml^−1^) and streptomycin (100 U ml^−1^), and maintained in a 5% CO_2_ humidified atmosphere at 37 °C. TZM-bl (JC53-bl) cells were obtained from the NIH AIDS Reagent Program, as contributed by John Kappes and Xiaoyun Wu (University of Alabama, Birmingham, AL) and maintained as above. TZM-bl is a HeLa cell clone genetically engineered to express human CD4, CXCR4 and CCR5, and to contain HIV *Tat*-responsive reporter genes for firefly luciferase (Luc) and β-galactosidase under regulatory control of the HIV-1 long terminal repeat^31^. MS40L cells^32^, kindly provided by Xin Luo (Virginia Polytechnic Institute, Blacksburg, VA), were maintained in Iscove's modified Dulbecco's medium plus 10% FCS and antibiotics, and used for B-cell feeder cultures. Expression of human CD40L by MS40L cells was confirmed by indirect immunofluorescence assay. All cell lines tested negative for mycoplasma.

### Transient stimulation of memory B cells

Cryopreserved PBMCs were thawed, washed and suspended in medium containing 10% FCS and antibiotics. B cells and/or memory B cells were enriched using either positive or negative selection with immunomagnetic beads (Miltenyi, San Diego, CA). In the early phases of our studies B cells were cultured at low cell densities (25–50 cells per well) in medium RPMI 1640 containing 10% FCS, 2 μg ml^−1^ R848 (InVivoGen, San Diego, CA) and 100 U ml^−1^ IL-2 in multiple 96-well culture plates seeded with irradiated mature human macrophages as feeder cultures^33^,^34^. In later experiments memory B-cell fractions were co-cultured at low cell densities in Iscove's modified Dulbecco medium containing 10% FBS, 2 μg ml^−1^ CpG2006, 100 U ml^−1^ rIL-2 and 25–50 ng ml^−1^ rIL-21, with MS40L feeder cells (5,000 cells per well) in multiple 96-well plates. MS40L was derived from a murine stem cell line (MS5) engineered to express low levels of cell surface human CD40L (ref. 32). When used as feeder cells MS40L cells support robust B-cell growth. Irradiation of MS40L cells was not necessary as these cells do not overgrow the cultures. In both culture systems, fresh medium containing growth factors was added after 7 days of culture and after each antibody-screening procedure. Screening for antibody production was begun at 12–14 days and was repeated at 3- to 4-day intervals as necessary. rIL-2 was obtained from the NIH AIDS Reagent Program as provided by Maurice Gately (Hoffmann–La Roche).

### Screening immunoassay

The ectodomain of LASV GPC (Josiah strain) lacking the transmembrane domain and intracytoplasmic tail (rGPe) was expressed in insect cells and purified by affinity chromatography and gel filtration. Purified LASV rGPe was stored in aliquots at 4 °C. The protein was used to coat wells of ELISA plates at 1.25 μg ml^−1^ in fresh 0.1 M NaHCO3 for 1 h at room temperature. Wells were washed and blocked with RPMI 1640 medium and 10% FBS for 30 min. Supernatant fluids from 96-well B-cell cultures were transferred to assay plates and incubated for 1 h at room temperature. The wells were again washed and incubated with peroxidase-conjugated goat anti-human IgG-Fc (Southern Biotech, Birmingham, AL) diluted 1:2,000 in PBS containing 0.5% Tween 20 and 5% whey protein (BiPro, Le Sueur, MN). After a final wash step, colour was developed by the addition of 3,3′,5,5′-Tetramethylbenzidine (TMB)-H_2_O_2_ as the substrate for peroxidase. Colour development was stopped after 3–4 min by the addition of 1 M phosphoric acid. Colour was read as absorbance (optical density) at 450 nm. In some experiments, the LASVpp reporter infectivity assay described below was used to screen B-cell cultures for the production of neutralizing antibodies.

### Antigen capture ELISAs and mAb competition assay

Wells of ELISA plates were coated for 1 h at room temperature with ConA (25 μg ml^−1^ in 0.1 M HEPES, pH 7.5) and incubated with cell lysates prepared in 1% Triton X-100 as previously described for dengue virus E proteins^33^. Wells were blocked with medium containing 10% FBS. mAbs were incubated in wells for 1 h at 5 μg ml^−1^. Thereafter, the ELISA was completed as in the screening immunoassay. For ELISA with D7-tagged GP1 mutants, we coated wells with the murinized mAb JR52, which binds D7. Wells were blocked with medium plus 10% FBS and reacted for 1 h with mAbs at 5 μg ml^−1^. Standard ELISA procedure was followed as described above.

For mAb competition assays, ConA-captured LASV GPC was incubated for 1 h with 50 μg ml^−1^ of each competing unlabelled mAb or buffer. Then, biotinylated mAbs diluted to predetermined optimal dilution were added to wells with vigorous mixing. After 1 h wells were washed and reacted another hour with peroxidase–streptavidin (Vector Labs, Burlingame, CA) diluted 1:1,000. Wells were washed and ELISA was completed as in the screening immunoassay.

### Peptide ELISA

Peptides were designed as overlapping 15-mers, spanning the entire length of the LASV Josiah GPC genome (61 peptides total). Each peptide contains one unique amino acid, with seven amino acids overlapping with the preceding peptide and seven amino acids overlapping with the following peptide. Peptides were synthesized by ProImmune (Oxford, UK) to 95% purity confirmed by liquid chromatography–mass spectrometry. Lyophilized peptides were resuspended in 40% acetic acid solution as 3 mg ml^−1^ stock solutions. Final acetic acid concentrations did not exceed 1% in assays. Nunc PolySorp 96-well plates (Sigma-Aldrich, St. Louis, MO ) were coated with peptides at a final concentration of 1 μg per well in 100 mM sodium bicarbonate coating buffer, pH 9.5. Plates were incubated overnight at 4 °C and washed three times with PBS-Tween. Plates were then blocked for 60 min with 250 μl of blocking buffer consisting of 5% milk in PBS-Tween, then washed as above. Each mAb was incubated in blocking buffer was added at a final volume of 100 μl to each well. The plates were incubated for 1 h at 37 °C, then washed as above. Detection was performed with 100 μl per well of horseradish peroxidase-conjugated goat α-human IgG antibody reagent (KPL)) diluted to 1:2,500 in blocking buffer. After 1 h incubation, 100 μl to each well of TMB-H_2_O_2_ substrate was added, and the plates were incubated for 5 min. The reaction was stopped by adding 100 μl per well of TMB stop solution and read at 450 nm.

### Antibody-capture ELISA

Arenavirus rGPe used in ELISA experiments was expressed in insect cells and purified by affinity chromatography and gel filtration. Corning Costar 96-well half-area assay plates (Corning Incorporated, Kennebunk, ME) were coated with 50 μl at a final concentration of 0.5 μg per well in PBS. Plates were incubated overnight at 4 °C and washed three times with PBS-Tween. Plates were then blocked for 1 h with 125 μl of blocking buffer consisting of 3% BSA/PBS, then washed as above. Each mAb was diluted to a final concentration of 5 μg ml^−1^ in blocking buffer was added at a final volume of 50 μl per well. The plates were incubated for 1 h at 37 °C, then washed as above. Detection was performed with 50 μl to each well of horseradish peroxidase-conjugated goat α-human IgG antibody (Southern Biotech) diluted to 1:2,500 in blocking buffer. After 1 h incubation, 50 μl to each well of TMB-H_2_O_2_ substrate was added, and the plates were incubated for 5 min. The reaction was stopped by adding 50 μl to each well of TMB stop solution and read at 450 nm.

### Cloning and expression of human immunoglobulin genes

Cells from wells testing positive for antibody in screening assays were collected, suspended either in RNAlater (Ambion, Austin, TX) or guanidine lysis buffer and stored frozen for later RNA extraction and purification (Ambion RNAqueous-Micro Total RNA Isolation Kit). We amplified VH, Vκ or Vλ genes in separate reactions from RNA purified from stored B cells using a one step RT–PCR (Invitrogen SuperScript III kit with Platinum Taq High Fidelity polymerase) with primer mixes designed to amplify the different families of heavy- and light-chain genes^35^. For expression vector assembly, forward primers included a 25 nucleotide non-annealing 5′-tag sequence, which was homologous to the immunoglobulin leader sequence at the 3′-end of the cytomegalovirus (CMV) promoter fragment. Reverse primers were designed to overlap the 5′-end of the immunoglobulin constant region for each vector. VH, Vκ or Vλ PCR products from each B-cell culture were inserted by overlapping PCR between two DNA fragments for the construction of full-length linear expression vectors. The upstream fragment contained the CMV promoter and the immunoglobulin leader sequence and the downstream fragment was made up of constant region sequences for IgG1, kappa or lambda genes followed by a C-terminal BGH poly A sequence. Paired heavy- and light-chain full-length linear expression vectors were co-transfected into 293T cells, and supernatant fluids of transfected cultures were tested for antibody activity in the same assays used to screen the B-cell cultures. This step allowed rapid detection of pairs of VH and VL chain genes that functioned together to produce antibody and also determined which light chain was involved.

To produce mAbs, we used the In-Fusion cloning system (Clontech, Mountain View, CA) to insert re-amplified pairs of VH and VL gene fragments into pLM2 expression plasmids similar to those described by Smith *et al*.^36^, but modified to contain the immunoglobulin leader sequence in the linear vectors^35^. First, expression plasmids were linearized by restriction enzymes acting on sites within the multiple cloning site of plasmid (EcoRI for IgG1 and lambda, and BsiWI for kappa). Linearized vectors were then PCR amplified with primer pairs designed to create terminal sequences that were homologous to 5′ and 3′ terminal sequences of the variable region insert fragments, allowing insertion of the VH and VL fragments into linearized plasmids by the activity of the In-Fusion enzyme according to the manufacturer's instructions. The resulting plasmids were transformed in JM109 cells. We used a previously described strategy to identify the correct pair of VH and VL clones responsible for antibody production^37^. Subsequent sequencing of multiple clones showed that only one heavy and one light chain were capable of directing mAb synthesis. mAbs encoded by each pair of immunoglobulin plasmids were produced by transient transfection of HEK293T cells. mAbs were purified by Protein A affinity chromatography.

### Indirect immunofluorescence assays

Two methods were used for testing LASV mAbs for binding to arenavirus GPCs by indirect immunofluorescence. In the first method, HEK293T cells were transfected with plasmids constructed to express full-length LASV GPC, or GP1 or GP2. In preliminary studies, we established that the use of air-dried, unfixed cell smears gave optimal preservation and exposure of epitopes of GPC allowing positive recognition by all mAbs in the panel and that no LASV mAb was reactive with untransfected HEK293T cells. Cells were collected at 36–40 h post transfection and mono-dispersed in PBS. Cell smears were made from slurries of cells applied in 1–2 μl volumes to wells of polytetrafluoroethylene printed 10 or 24 well multi-test slides (Electron Microscopy Sciences, Hatfield, PA). Cell smears were air-dried for at least 10 min, blocked briefly with RPMI-10% FCS and incubated with 20 μl of mAbs diluted in RPMI-10% FCS for 30–45 min at room temperature in a moist chamber. Slides were washed in PBS and then incubated with fluorescein isothiocyanate (FITC)-anti-human IgGFc (1:50 dilution, Southern Biotech, Birmingham, LA) for 45 min. Slides were again washed with PBS, mounted with Vectashield under glass coverslips and examined under a Nikon epifluorescence microscope with × 40 objective.

In the second method, HEK293T cells transfected in 48-well culture plates with GPC-expressing plasmids were fixed for 15 min in 4% formaldehyde, permeabilized with 0.2% Triton X-100 and blocked with 2.5% bovine serum albumin. Fixed-cell monolayers were then incubated with LASV mAbs, or with the following control murine mAbs: 36.1 (LCMV GP-1)^38^; L52-74-7A (LASV GP-1)^39^; or 83.6 (OW arenavirus cross-reactive GP-2)^20^ for 1 h at room temperature. Slides were washed with PBS and then incubated with either FITC-conjugated anti-human (1:250 dilution, Jackson ImmunoResearch, West Grove, PA) or FITC-conjugated anti-mouse (1:140 dilution, Dako) secondary antibodies for 1 h at room temperature. After three washes with PBS, cells were examined under a Leica fluorescence microscope and photographed (Cooke Sensicam QE) with a × 20 objective.

### Immunoprecipitation

Cryopreserved GPC-expressing cells were thawed, washed and suspended in buffer contain 0.1% BSA. In one immunoprecipitation format, mAbs were incubated for 30 min with clarified lysates of cells expressing GPC prepared either in RIPA buffer (Sigma # R0278 containing 150 mM NaCl, 1.0% IGEPAL CA-630, 0.5% sodium deoxycholate, 0.1% SDS and 50 mM Tris (pH 8.0)) or 1% Triton X in PBS. Halt Protease Inhibitor Cocktail (Pierce, Rockford, IL) was added to the lysis buffers and immune complexes were captured on magnetic protein A beads (Pierce, Rockford, IL or Thermo-Fisher, Waltham, MA).

In a second format, mAbs were first captured on Protein A magnetic beads and washed mAb-bound beads were then incubated 30 min with RIPA or Triton X lysates of GPC-expressing cells. In both methods beads containing bound immune complexes were washed 8–10 times in round bottom 96-well plates with RIPA buffer containing 0.2% SDS by repeated capture on an EpiMag HT (96-Well) Magnetic Separator (Epigentek, Farmingdale, NY) and vigorous resuspension in RIPA buffer on a Genie plate shaker (VWR, Radnor, PA).

Beads in each well were suspended in 50 μl NuPage Sample buffer without reducing agents and heated 10 min at 75 °C to elute bound proteins. Immunoprecipitated proteins were resolved in duplicate gels by electrophoresis in pre-cast NuPAGE 10% Bis–Tris gels, according to the manufacturer's specifications (Novex, San Diego, CA). Proteins from each gel were electrophorectically transferred to nitrocellulose membranes in the iBlot Dry Blotting (Thermo-Fisher, Walthan, MA) apparatus. In some experiments, Protein A beads with captured immune complexes were exposed to RIPA buffer containing 1.5 M NaSCN before the bound complexes were eluted for PAGE and western blotting analysis. Duration of exposure to NaSCN varied from 1 h to overnight.

Proteins eluted from Protein A beads in immunoprecipitation experiments were resolved by SDS–PAGE in 10% NuPAGE gels and transferred to nitrocellulose in XCell blot modules (Invitrogen, Carlsbad, CA). Blotted membranes were blocked for 1 h or overnight in PBS containing 5% non-fat dry milk and placed in a MPX multi-channel blotter apparatus. mAbs at 5 μg ml^−1^ in 5% milk containing 0.5% Tween-20 were incubated in individual channels for 1 h. For each pair of blotted membranes, one was incubated 1 h with a mixture of murinized mAbs specific for LASV GP1 at 2 μg ml^−1^; the other was incubated for 1 h with a mixture of murinized mAbs specific for LASV GP2 at 2 μg ml^−1^. Blots were washed with PBS-0.5% Tween and incubated for an additional hour with 1:10,000 dilution of IRDye 680RD Goat anti-mouse IgG (H+L) (LI-COR Biosciences, Lincoln, NE). The membranes were then washed extensively and scanned in and Odyssey Infrared Imager (LI-COR Biosciences, Lincoln, NE).

Images in Fig. 3 and Supplementary Fig. 5 show that results of the immunoprecipitation experiments have been cropped and lanes have been rearranged for clarity of presentation. Full-size western blot images containing all lanes in their original order are presented in Supplementary Fig. 6.

### Conversion of human mAb IgG1 heavy chains to murine IgG2a

To create reagents for probing western blots from immunoprecipitation experiments we replaced the human heavy-chain constant regions of several human IgG1 mAbs with murine IgG2a heavy chains. This allowed use of anti-murine IgG secondary antibody reagents without cross-reactivity with the human IgG mAbs used in immunoprecipitation. The murine IgG2a constant region in an expression plasmid, kindly provided by Yongjun Guan (University of Maryland), was PCR amplified and the PCR product was then inserted into linearized human mAb heavy-chain expression vectors. These vectors were subjected to a PCR, which in effect removed the human IgG constant region and created terminal sequences that overlapped the nucleotide sequences of the murine IgG2a insert. Following the methods described by Benoit *et al*.^40^ we inserted the murine IgG2a PCR product into the linearized vector using the In-Fusion ligase independent cloning method (Clontech, Mountain View, CA).

### Expression of recombinant proteins

Eukaryotic expression vectors based on pCAGGS encoding full-length cDNAs of LASV lineage IV (Josiah, NP_694870) and LCMV GPCs were previously described by Rodrigo *et al*.^41^. Similar plasmids for expression of GPC from LASV lineage I (LP strain, CDC specimen 812285, AAF86701), LASV lineage II (803213 strain; CDC specimen 810801, AAF86703), LASV lineage III (GA391 strain; CDC specimen 806791, P17332), Mobala virus (MOBV, YP_516226), Mopeia virus (MOPV, YP_170705), Merino Walk virus (MEWV, YP_009019200) and LUJV (CDC specimen 812096, NC_012776) were generated by cloning full-length cDNA encoding each GPC into the pCAGGS plasmid. Each cDNA was PCR amplified from RNA extracted from infected cells and inserted into the pCAGGS plasmid. In addition, full-length GPC cDNA from LASV Josiah strain and three other LASV strains were cloned into pLM2, a PBR322-based vector, which we routinely use for mAb heavy- and light-chain expression as described above. Arenavirus rGPe (LCMV HPI AJ297484.1, LUJVNC_012776 and MACV Carvallo AY571904) used in other ELISA experiments was expressed from synthetic genes in insect cells and purified by affinity chromatography and gel filtration.

### Mutagenesis of LASV glycoproteins

Using site-directed mutagenesis, we constructed a series of mutants of full-length LASV GPC Josiah in which progressively longer segments of GP2 from the N terminus were deleted, while leaving GP1 intact. Deletion mutants that removed the N-terminal 16 amino acids of GP1 (aa60–75), or deleted the fusion loop (αα280–291), the HR1 regions (αα311–355), the T-loop (αα365–384), or part of HR2 (aa400-412) in GP2, were similarly constructed. Triple-alanine substitutions were also introduced by PCR mutagenesis into the N terminus of GP1 in the full-length GPC vector from residues K63 through N93.

A plasmid (pLM2-GP1-D7) for expression of GP1 alone was created by deletion of GP2 (aa260–491) from full-length GPC and replacing it with the D7 epitope tag (APTKAKRRVVQREKR), representing the conserved C terminus of HIV-1 gp120 (ref. 42). This epitope is recognized by sheep antibody D7324 (Aalto Scientific), which is widely used in gp120 capture ELISAs in HIV studies^43^ and is also recognized by a murinized mAb, JR-52, described below. HEK293T cells transfected with SP-GP1-D7 efficiently produced secreted GP1, which was quite stable. We used this plasmid to introduce a series of single-alanine substitutions into GP1. Primers used for PCR mutagenesis were designed along the principles described by Liu and Naismith^44^. The presence of the correct mutations was confirmed by sequencing.

Plasmids expressing GPC with these mutations were transfected in 293T cells, and mAbs were tested for binding to ConA-captured glycoproteins from Triton X lysates of transfected cells. A standard panel of neutralizing and non-neutralizing mAbs was tested, which allowed an assessment of the overall expression levels of each mutant. A deletion or mutation that reduced binding of mAbs by >90% compared with binding to unmutated control GPC was considered to have a critical effect. To assure that the deletions did not induce global structural effects, it was also necessary to show that several groups of non-neutralizing mAbs maintained normal or minimally affected binding activity to each mutant compared with unmutated GPC.

To test mAb binding to GP1-D7 and alanine mutants, we coated wells with a murinized rhesus mAb, JR52 (a gift of Yongjun Guan, University of Maryland), which binds the D7 epitope. mAbs were incubated with JR52-captured GP1 or mutant GP1, and their binding was assessed with a peroxidase-conjugated goat anti-human IgGFc in a standard ELISA format.

### Pseudovirus assay

We prepared Lassa pseudoviruses (LASVpp) capable of a single round of replication by co-transfection of HEK293T cells with LASV GPC plasmids and pSG3Δenv, encoding the envelope-deficient core of HIV-1 (ref. 31). These LASVpp are readily assayed in TZMbl cells^31^,^45^, a HeLa cell derivative that contains integrated luciferase and β-galactosidase genes under regulatory control of an HIV-1 LTR, which is activated by HIV-1tat after virion entry. The method to produce pseudoparticles was the same as used previously for generating HIV-1 pseudoviruses^46^. HEK293T cells were plated in T75 flasks and grown overnight before transfection with 10 μg of LASV GPC expression plasmids and 10 μg of pSG3. Supernatant fluids containing LASVpp were collected at 72 h after co-transfection, clarified by centrifugation, filtered and frozen in aliquots −80 °C for later use. For determination of pseudovirus titres, monolayers of 50,000 TZMbl cells per well in black 96-well plates were infected with serial dilutions of virus stocks. Luciferase activity in the cultures was measured at 48 or 72 h after inoculation using a commercially available kit (Promega BriteGlo). Luminescence was measured with a Wallac 1420 Multilabel Counter (PerkinElmer, Waltham, MA).

The neutralizing activities of purified mAbs were determined by incubating mixtures of LASVpp stocks at predetermined optimal dilutions with serial dilutions of each mAb. The assay was then performed exactly as discussed above, and the per cent inhibition of viral replication at each antibody dilution was determined. We also used the LASVpp assay to screen for neutralizing antibodies. This involved incubating supernatant fluids from B-cell cultures with LASVpp in black 96-well culture plates. TZMbl cells were added to a final density of 5 × 10^3^ cells in each well and the plates were incubated for 48–72 h. Neutralization was assessed by analysing the amount of luciferase produced from the pseudovirus-infected cells. Neutralization activity was defined as >50% reduction in luciferase activity after a single-round infection.

### LCMV-based pseudoparticle preparation

In a second system LASV or LCMV GPC was pseudotyped with rLCMV GPC/GFP^41^. Vero and BHK-21 cell lines stably expressing LCMV or LASV GPCs were maintained in complete media supplemented with hygromycin B (250 μg ml^−1^). Single-cycle infectious recombinant LCMV (Armstrong strain; ARM5-3b) expressing the green fluorescent protein, GFP (rLCMVΔGPC/GFP) pseudotyped with LCMV or LASV GPC (pLCMV or pLASV, respectively) were propagated in BHK-21 cells constitutively expressing LCMV or LASV GPC by infecting at multiplicity of infection of 0.01 and collecting the tissue culture supernatants at 72 h post infection. Virus titres of stocks were determined by immunofocus assay (fluorescent forming units) in GPC-expressing Vero cells.

Microneutralization assays using the pLASV and pLCMV have been previously described^41^. Briefly, a predetermined amount of virus to obtain 75–100% GFP-positive cells at 48–72 h post infection was pre-incubated with twofold dilutions of the LASV mAbs at 37 °C for 90 min. LCMV GPC-expressing Vero cells (96-well plate format, 10^4^ cells per well, triplicates) were then infected with the virus/antibody mix at 37 °C for 90 min. The immune complexes were then removed and infection was maintained in normal media at 37 °C. At 48–72 h post infection, cells were monitored for GFP expression using a fluorescent microscope. GFP intensity was measured with a microplate reader (Beckman Coulter, Pasadena, CA). Virus inoculum in the absence of LASV mAbs was used as negative control to set up 100% GFP expression. Per cent neutralization was fitted on a sigmoidal dose–response curve using the GraphPad Prism software and used to calculate the EC_50_ of each LASV mAb.

### Plaque reduction neutralization test

To assess neutralizing activity of mAbs, a standard PRNT with infectious LASV was performed under BSL-4 conditions^47^. A standard amount of LASV (∼100 plaque-forming units) was incubated for 60 min with serial twofold dilutions of each mAb in Earle's Minimal Essential Medium, 1% Pen/Strep and 1 × glutamax containing 10% guinea pig complement (Rockland, Limerick, PA). After incubation, these reaction mixtures were assayed for residual infectivity as measured by plaque-forming units^47^. The mixture was used to inoculate Vero 76 cells for 60 min. The inoculum was removed and cells were overlaid with an agar medium and incubated for 4–6 days at 37°C. Cells were stained with neutral red for 24 h and plaques were counted. End-point titres were determined through standard 4-parameter logistic regression analysis and represented as the dilution of antibody, which neutralized 50% of the plaques. For these assays we used a low-passage LASV Josiah virus stock, which was kindly provided by Tom Ksiazek (University of Texas Medical Branch-Galveston, TX), and originated from CDC Lassa-Josiah CDC number 057562. This strain was originally isolated from a human clinical specimen that was passed once in Vero cells and twice in Vero E6 cells.

### Modelling of LASV GPC

SWISS-MODEL (http://swissmodel.expasy.org/), an automated system for homology modelling the three-dimensional structure of a protein from its amino-acid sequence^48^, was use to thread aa59–424, which comprise the ectodomain of LASV GPC (Josiah strain, lineage IV), on to the recently determined structure of LCMV GPe^14^. The final LASV GPe model has an RMSD of 0.84 as compared with LCMV GPe with a GMQE of 0.81 (Global Model Quality Estimation where identical structures have a GMQE of 1). Location and orientation of the N-linked glycan were modelled based on the location of the cognate glycan in the LCMV GPe structure template. The exception to this is the glycan at N99, which does not exist in LCMV GPe. Instead, this glycan was modelled using Coot^49^ to minimize clashes with surrounding residues.

To assist sequence alignments we used PredictProtein (https://www.predictprotein.org), a meta-service that provides multiple sequence alignments, and predicts aspects of protein structure such as secondary structure, solvent accessibility, transmembrane helices and strands, coiled-coil regions, disulfide bonds and disordered regions. PyMOL (https://www.pymol.org), an open-source, user-sponsored, molecular visualization system created by Warren Lyford DeLano and currently commercialized by Schrödinger, Inc., was used to produce three-dimensional images.

### *In silico* analysis of antibody sequences

Antibody heavy and light-chain constructs were sequenced by the Sanger method (Agencourt Bioscience Corporation, Beverly, MA). Sequence accuracy was verified using multiple independent clones for each unique heavy and light chains. Consensus immunoglobulin sequences spanning framework 1 through 4, and encompassing CDRs 1–3 were subjected to *in silico* analysis using the IMGT/V-QUEST online database search and analysis software package, reference directory release 201515-3 (8 April 2015), employing a full complement of analytical parameters and compared with the human (*Homo sapiens*) immunoglobulin set^50^. Additional analyses were performed using the abYsis integrated antibody sequence analysis and prediction Software package (http://www.bioinf.org.uk/abysis/). The analysis reported in this manuscript used the Kabat numbering scheme to derive and characterize framework and CDR domains in antibody sequences.

### Kinetics of LASV mAb binding

mAb-binding analysis done on a Biacore 3000 (GE Healthcare, Little Chalfont, United Kingdom). A GE Healthcare Sensor Chip CM5 was coated with 15 μg ml^−1^ anti-FLAG antibody (Sigma-Aldrich, St. Louis, MO). Interaction analysis was performed by injection of LASV recombinant glycoproteins or FLAG protein (Sigma-Aldrich, St. Louis, MO) at concentrations that produced a response between 50 (*R*_min_) and 185 (*R*_max_) ‘response units'. Flow was 5 μl min^−1^ in a HEPES-EP buffer, pH 7.5. Once the proteins were covalently immobilized, the flow rate was changed to 15 μl min^−1^. Varying concentrations of LASV mAbs were then injected and kinetic parameters determined using a bivalent binding curve.

### Statistical analysis

Categorical data were summarized as frequencies and percentages (a) and continuous data were summarized as medians and their respective interquartile ranges (b–d). Data were considered in their individual form for those cases where monotonic trend was of primary interest. Statistical comparisons of categorical variables (a) were carried out using Fisher's exact test, and those comparisons for continuous variables (b–d) were carried out using the Wilcoxon Mann–Whitney test. Kendall's tau test for trend was used to assess monotonic trends in individual data (e). Analyses were two-tailed and carried out using the SAS System (version 9.3; SAS Institute, Inc., Cary, NC). *P* values were expressed in their exact forms, and the significance threshold was set at *P*<0.05.

## Additional information

**How to cite this article:** Robinson, J. E. *et al*. Most neutralizing human monoclonal antibodies target novel epitopes requiring both Lassa virus glycoprotein subunits. *Nat. Commun.* 7:11544 doi: 10.1038/ncomms11544 (2016).

## Supplementary Material

Supplementary Information Supplementary Figures 1-8, Supplementary Table 1, Supplementary Note and Supplementary References
