# Supplementary material for: Most neutralizing human monoclonal antibodies target novel epitopes requiring both Lassa virus glycoprotein subunits
Source: Nat Commun. 2016 May 10;7:11544. doi: 10.1038/ncomms11544 (PMC4866400; doi:10.1038/ncomms11544)
Supplement: Supplementary Information — Supplementary Figures 1-8, Supplementary Table 1, Supplementary Note and Supplementary References [file ncomms11544-s1.pdf]

SUPPLEMENTARY INFORMATION

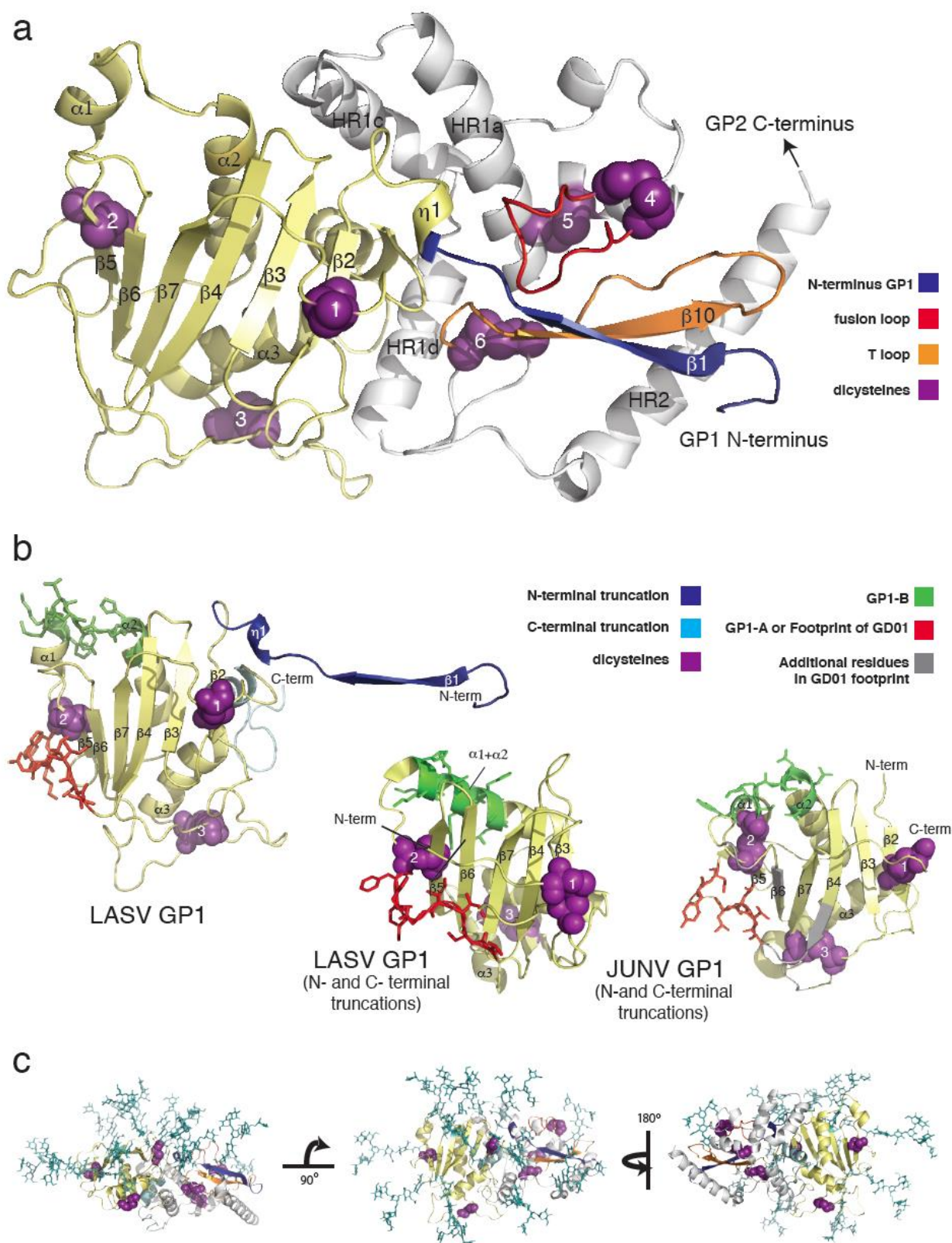

Supplementary Figure 1. Structural model of the LASV GPC ectodomain. LASV GPC was threaded onto the recently solved structure of the LCMV GPC ectodomain. **Panel a:** Homology model of LASV GP

ectodomain annotated according to Hastie *et al.* ¹. The model is colored to highlight the interaction of the N-terminus of GP1 (blue) with the fusion loop (red) and T-loop (orange) of GP2. Cysteine pairs are shown as purple spheres. The role, if any, of additional aa residues in GPC, including membrane proximal aa, the transmembrane domain, the intracytoplasmic domain and the SSP, in antibody binding is unknown. The SSP is known to interact with the intracytoplasmic domain to form a zinc-binding domain ². **Panel b:** Comparison of the modeled LASV GP1 structure (aa61-258), the structure of a rGP1 (aa81-237) containing truncations at the N-terminus (blue) and C-terminus (teal) as determined by Cohen-Dvashi *et al.*, ³ and the structure of a rGP1 (aa 87–235) containing similar truncations at the N-terminus (blue) and C-terminus (teal) as determined by Mahmutovic *et al.*, ⁴. The putative epitope recognized by non-neutralizing GP1-B MAbs consists of alpha helix 1 ($\alpha 1$) and alpha helix 2 ($\alpha 2$), which are separated by a loop in the LCMV GP ectodomain ¹ JUNV GP1 ⁴ and the LASV model, but are a single helix ($\alpha 1 + \alpha 2$) in the N- and C-terminal truncated GP1 structure determined by Cohen-Dvashi *et al.*, ³. The binding of some GP1-B MAbs, but not others is reduced by a N-terminal truncation of GP1 (Supplementary Figure 7a and unpublished data). The footprint of a neutralizing mouse MAb overlaps the putative GP1-A epitope. It is also interesting to note that the GP1-A MAb12.1F binding was not affected in by an N-terminal deletion in a GP1-only construct, but is affected by the same deletion in GPC. **Panel c:** Putative location of N-linked glycans on three views of the LASV GP ectodomain structure model. As in the LCMV GP ectodomain, all glycosylation sites are located on the upper face of the modeled LASV glycoprotein ectodomain.

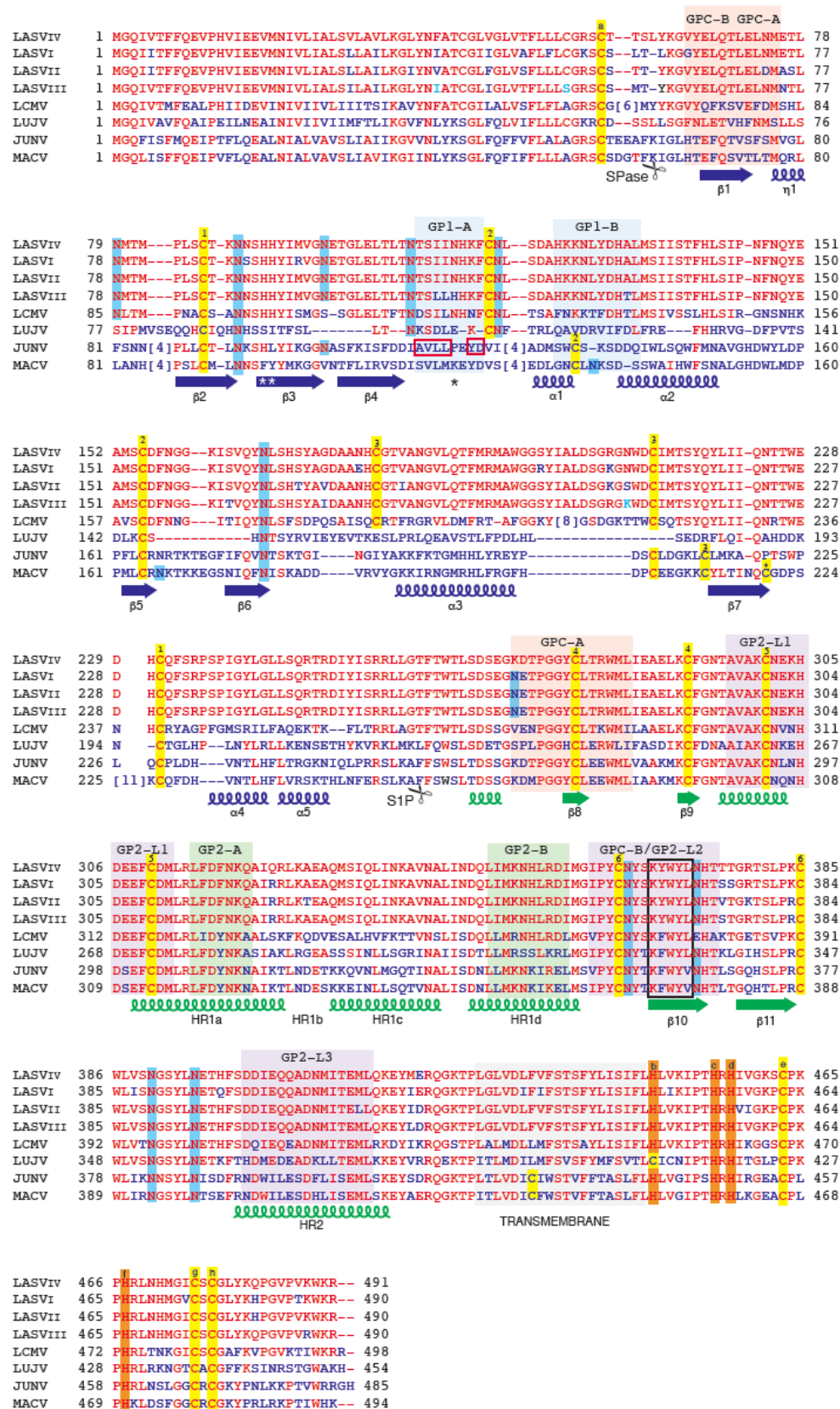

IV (Josiah strain). Residues in other sequences (LASV Pinneo lineage I], LASV 237-S-Nigeria-2010H lineage II, LASV Nig08-A19-S-Nigeria-2008H lineage III, LCMV HP1, LUJO, JUNV strain MN-2) and MACV Carvalho) that are different are depicted in blue. Structural features, including β -sheets (blue or green arrows), α -helices (blue or green helical symbol) and cysteine pairs (numbered yellow highlights) are annotated according to Hastie *et al.* ¹. Cysteine 220 (asterisk) is paired with cysteine 229 (not shown). Signal peptidase (SPase) and Ski protease (SK1) cleavage sites are indicated by a scissors symbol. Sequential runs of mostly hydrophobic residues that are likely to constitute the transmembrane anchor are highlighted in grey. Cysteine (yellow highlight) and histidine (orange highlight) residues in SSP and the C-terminal intracellular domain, which follows the transmembrane domain and coordinates with zinc atoms, are indicated by lower case letters ². Putative epitopes are indicated by light red, blue or purple highlights. Black asterisk: N119, the site of a neutralization escape mutant to murine MAb GP1-A⁵. Two white asterisks: histidines at aa92 and 93 in LASV GPC, which have been implicated in LAMP1 receptor binding by GP1 ³. Red boxes: Residues in JUNV GP1 that overlap with the footprint of neutralizing mouse MAb GD01 and the putative GP1-A epitope ⁴. Black box: Minimal epitope recognized by mouse MAb 33.6, which showed broad cross-reactivity similar to GP2-L1 MAbs 13.4E and 1.1B ⁶.

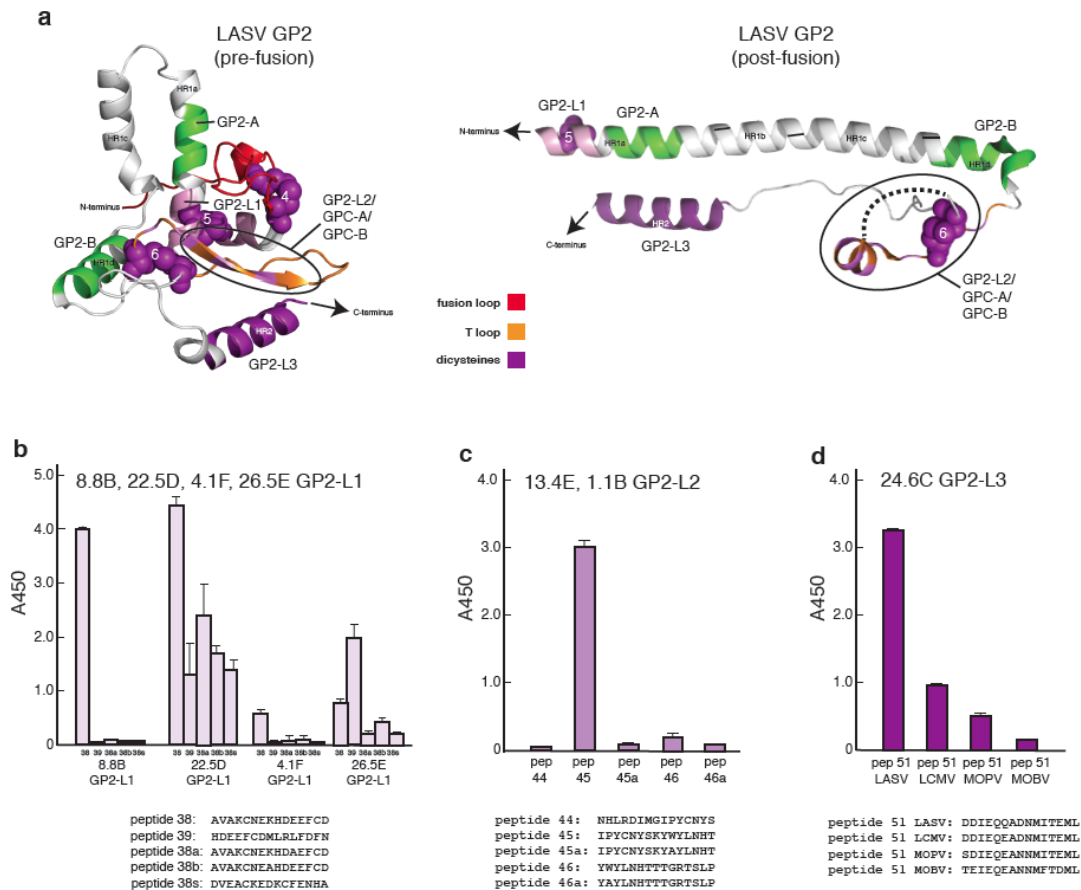

Supplementary Figure 3. Putative linear epitopes recognized by LASV MAbs. The panel of 113 LASV MAbs was tested by ELISA for binding to 61 overlapping synthetic 15-mer peptides designed to span the entire length of LASV GPC. **Panel a:** Location of the analogous sequences of peptides reactive with the indicated groups of MAbs on the structural model of LASV GP2 in the prefusion configuration is based on the recently determined structure of the LCMV glycoprotein ectodomain¹. Also shown are the locations of the putative GP2-A and -B epitopes and part of the putative GPC-B epitope. Shown for comparison is the modeled structure of LASV GP2 in the post-fusion configuration based on post-fusion LCMV GP2⁷. Seven MAbs reacted to peptides representing three linear epitopes in GP2. Four MAbs (4.1F, 22.5D, 25.6E, 8.8B) bound to peptides 38 AVAKCNEKHDEEFCD and/or 39 HDEEFCDMLRFDNF representing a linear epitope between the fusion loop and the HR1 region (aa296-311). Two MAbs (13.4E and 1.1B) bound to peptide 45 representing a linear epitope located in the T-loop and one MAb (24.6C) bound to peptide 51 DDIEQQADNMITEML (aa400-412) analogous to a portion of the HR2 region of GP2. MAbs 13.4E and 1.1B react to the same linear epitope CNYSKYWYLNHTTT (aa361-375) as murine MAbs 33.6 and 9-7.9 described previously⁶. The T-loop is also a part of the putative GPC-B epitope. Upon conversion to the post-fusion configuration of GP2 the loop between HR1a and HR1c is converted to an α -helix (HR1b). The β -sheet in the prefusion configuration of the T-loop undergoes a conversion in the post-fusion configuration to an α -helix. **Panel b:** Reactivity of GP2-L1 MAbs with variant peptides. 8.8B, 22.5D and 4.1F reacted more strongly to peptide 38 compared to peptide 39. 26.5E reacted more strongly to peptide 39, suggesting that T-loop contains more than one overlapping linear epitope. MAb 22.5D retained reduced reactivity to a peptide in which a lysine (K304) was changed to alanine, to a peptide in which a glutamic acid (E307) was changed to alanine, as well as to a scrambled peptide. Binding of 8.8B, 4.1F and 26.5E was nearly completely eliminated by these changes. **Panel c:** MAb 13.4E bound to peptide 45 but weakly to adjacent peptides 44 or 46. Alteration of the tryptophan (W370) residue in peptide 45 and 46 reduced strong binding to peptide 45 and the weak binding to peptide 46. **Panel d:** Immunofluorescence analysis presented in Supplementary Figure 6 below demonstrate that 24.6C reacts with LASV GPC, but did not react to LCMV, MOPV or MOB GPC. Consistent with this observation, peptides, analogous to peptide 51 of LASV GPC representing the similar sequences in GPC of LCMV, MOPV or MOB GPC, showed reduced reactivity to 24.6C.

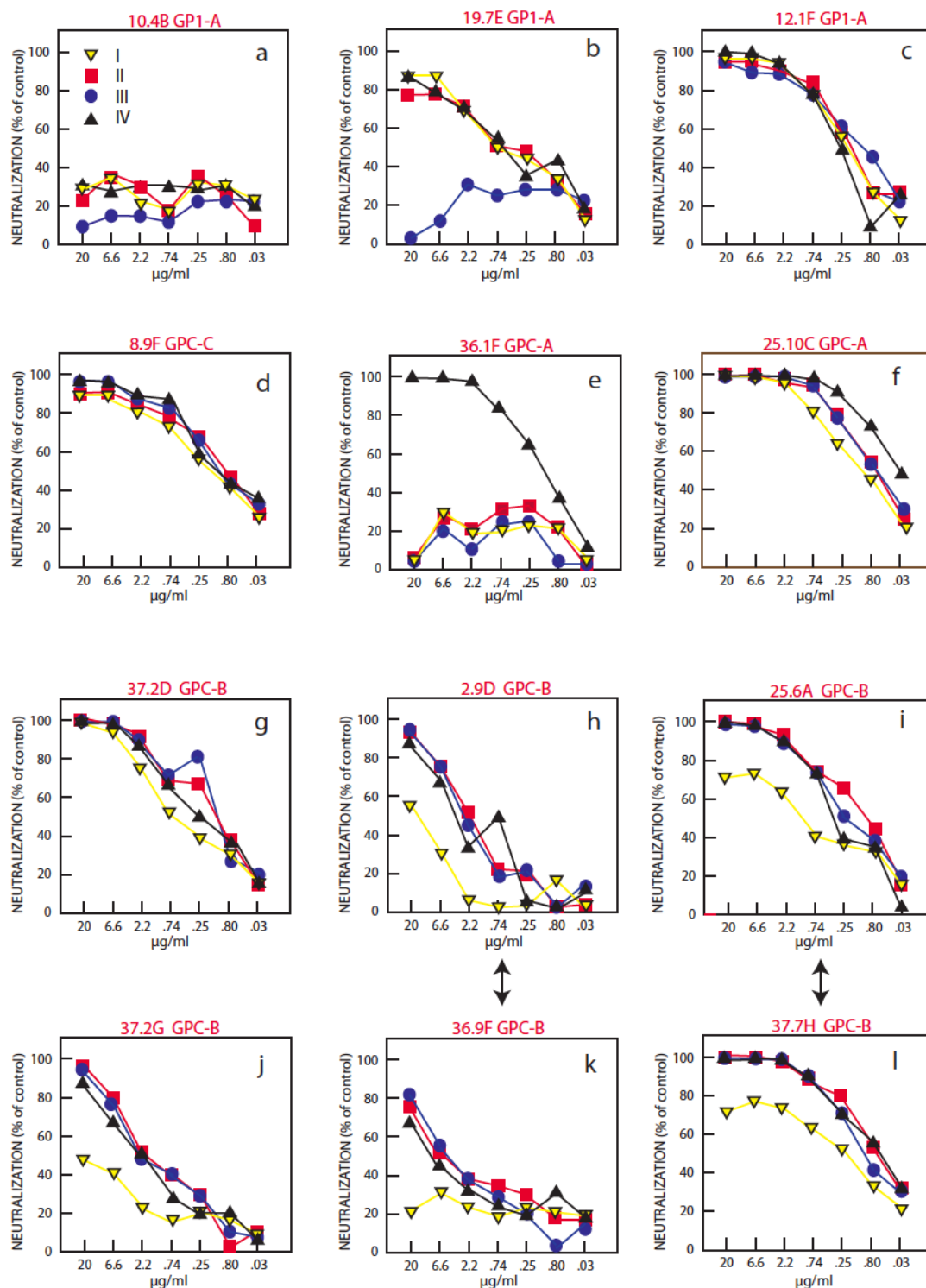

Supplementary Figure 4. Neutralization of LASV pseudoparticles expressing GPC of four lineages. The neutralizing activities of purified MABs were determined by incubating mixtures of LASVpp with an HIV-1 core with MABs at the indicated concentrations. 10.4B demonstrated low activity in PRNT, but limited to no neutralizing activity in the LASVpp assay. 12.1F, 8.9F and 37.2D had similar neutralizing activity against LASVpp expressing GPC of each of the 4 distinct LASV lineages. 19.7E has limited to no activity against LASVpp expressing lineage III GPC. 36.1 only neutralized LASVpp expressing lineage IV GPC. Antibodies 2.9D, 25.6A, 37.2G, 36.9F and 37.7H neutralized LASVpp expressing lineage IV, II and III GPC to similar extents, but had somewhat reduced neutralizing activity against LASVpp expressing lineage I GPC. The double-headed arrows indicate MABs with similar CDRs (Supplementary Table 1).

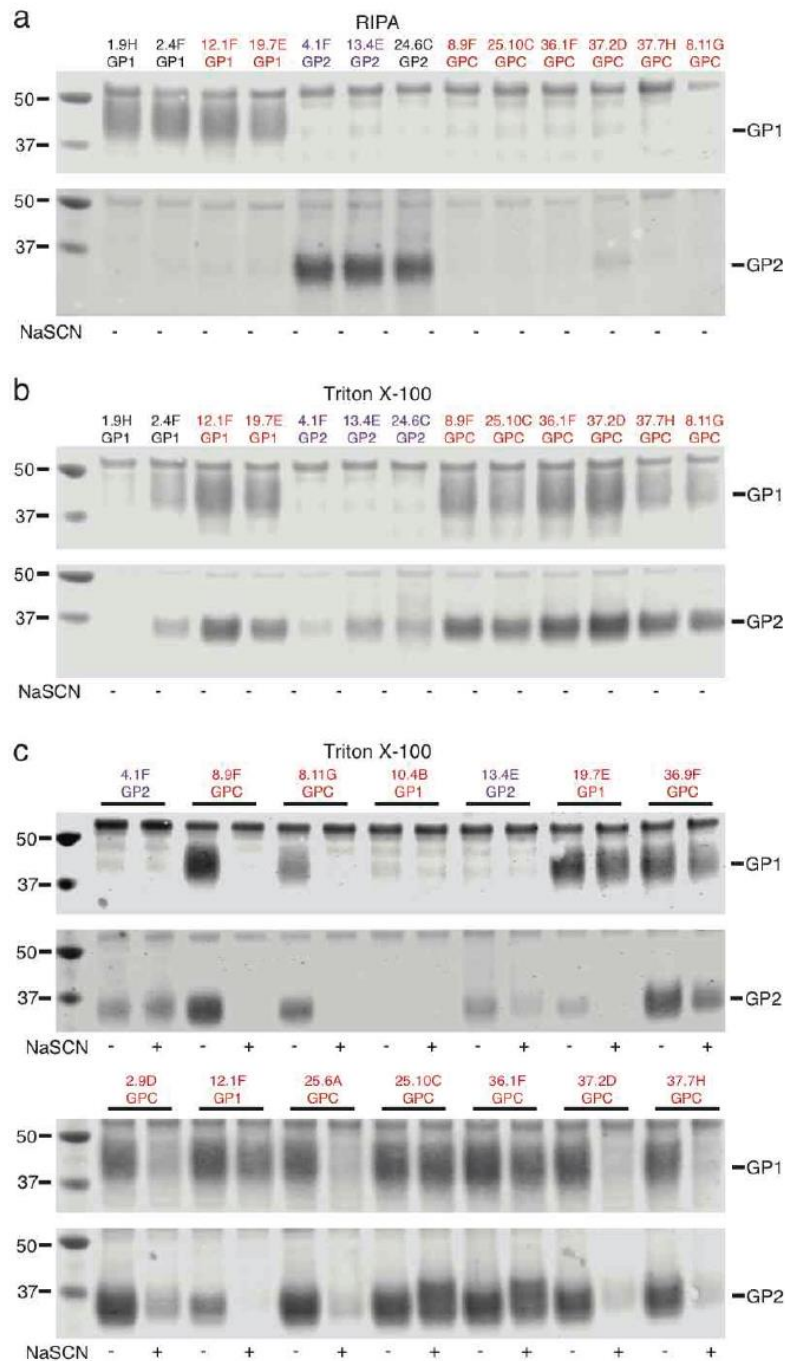

Supplementary Figure 5. Immunoprecipitation with anti-GPC MAbs in RIPA buffer, Triton X-100 and Triton X-100 ± NaSCN. LASV MAbs indicated were used as capture antibodies in immunoprecipitation studies employing lysates from cells expressing LASV GPs (GPC, rGP1 and rGP2). Immunoprecipitated proteins were resolved by SDS-PAGE, transferred to nitrocellulose and western blotting was performed with mouse monoclonal antibodies to either GP1 (upper gel image in each panel) or GP2 (lower image). **Panel a:** Immunoprecipitation with the indicated MAbs was performed with cell lysates prepared in RIPA buffer. **Panel b:** Immunoprecipitation was performed with cell lysates prepared in Triton X-100 containing buffer. **Panel c:** Immune complexes were formed between MAbs and LASV glycoproteins in Triton X-100 lysates in the presence or absence of NaSCN then immunoprecipitated with the indicated MAbs. Spliced, reordered images

of these gels showing a subset of the MAbs tested are presented in Fig. 3. Uncropped blots are shown in Supplementary Fig. 6. Experiments were repeated at least twice.

Full length Western blot data shown in Figure 3a and 3b and Supplementary Figure 5a and 5b

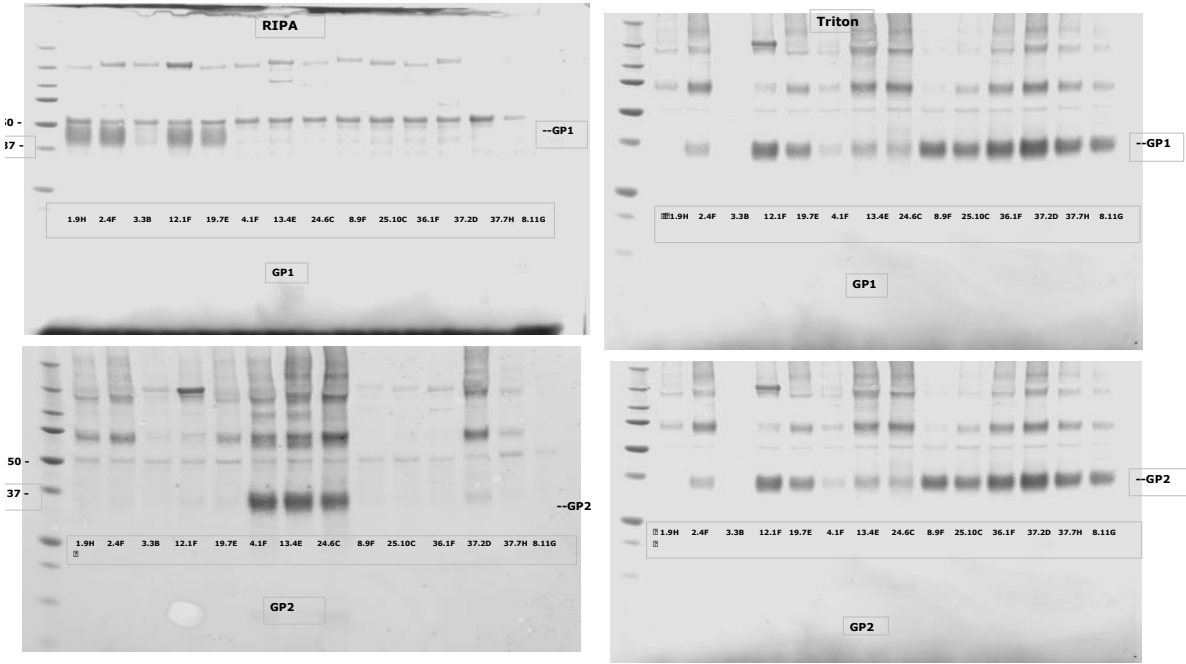

Full length blots of data shown in Figure 3C and Supplementary Figure 5c

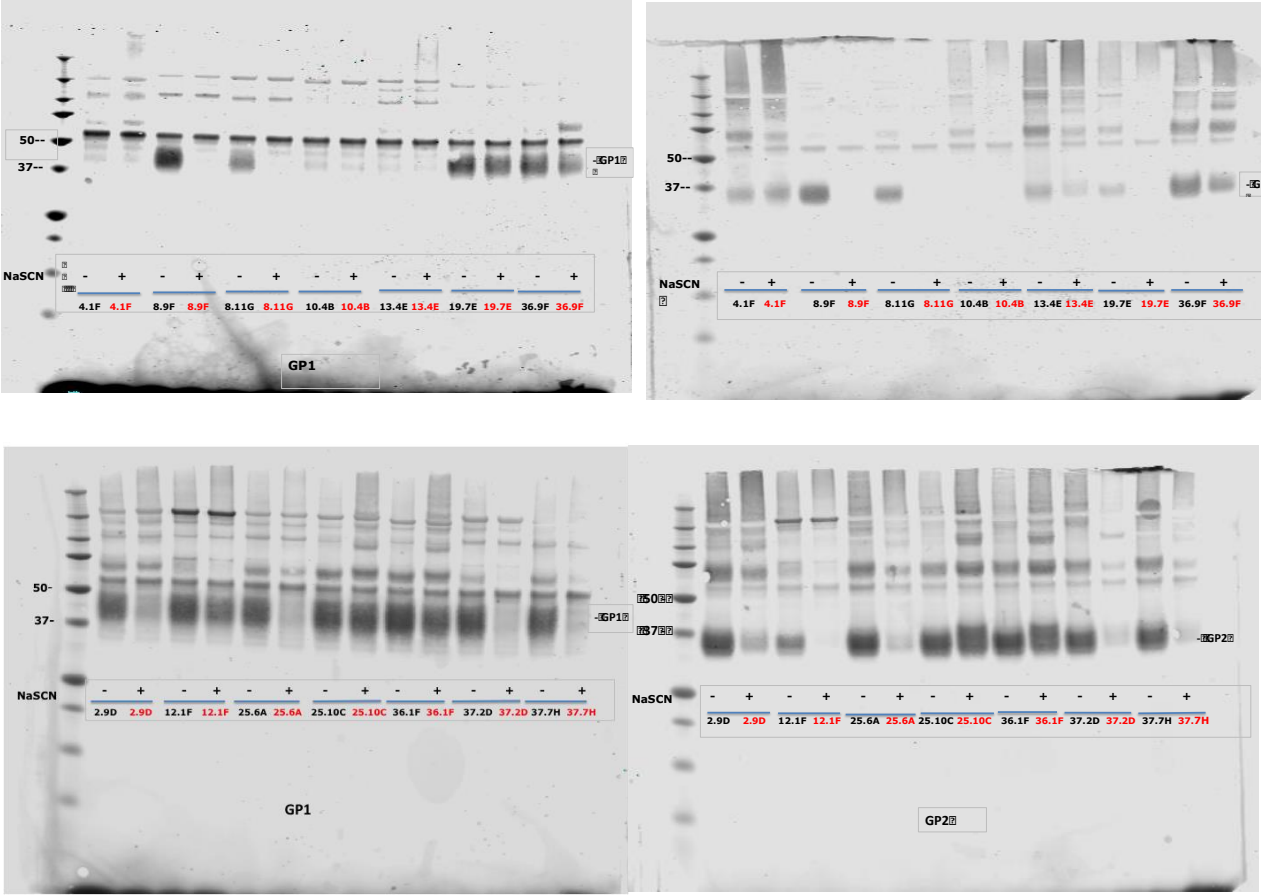

Supplementary Figure 6. Uncropped Western blots used to prepare Fig. 4 and Supplementary Fig. 5.

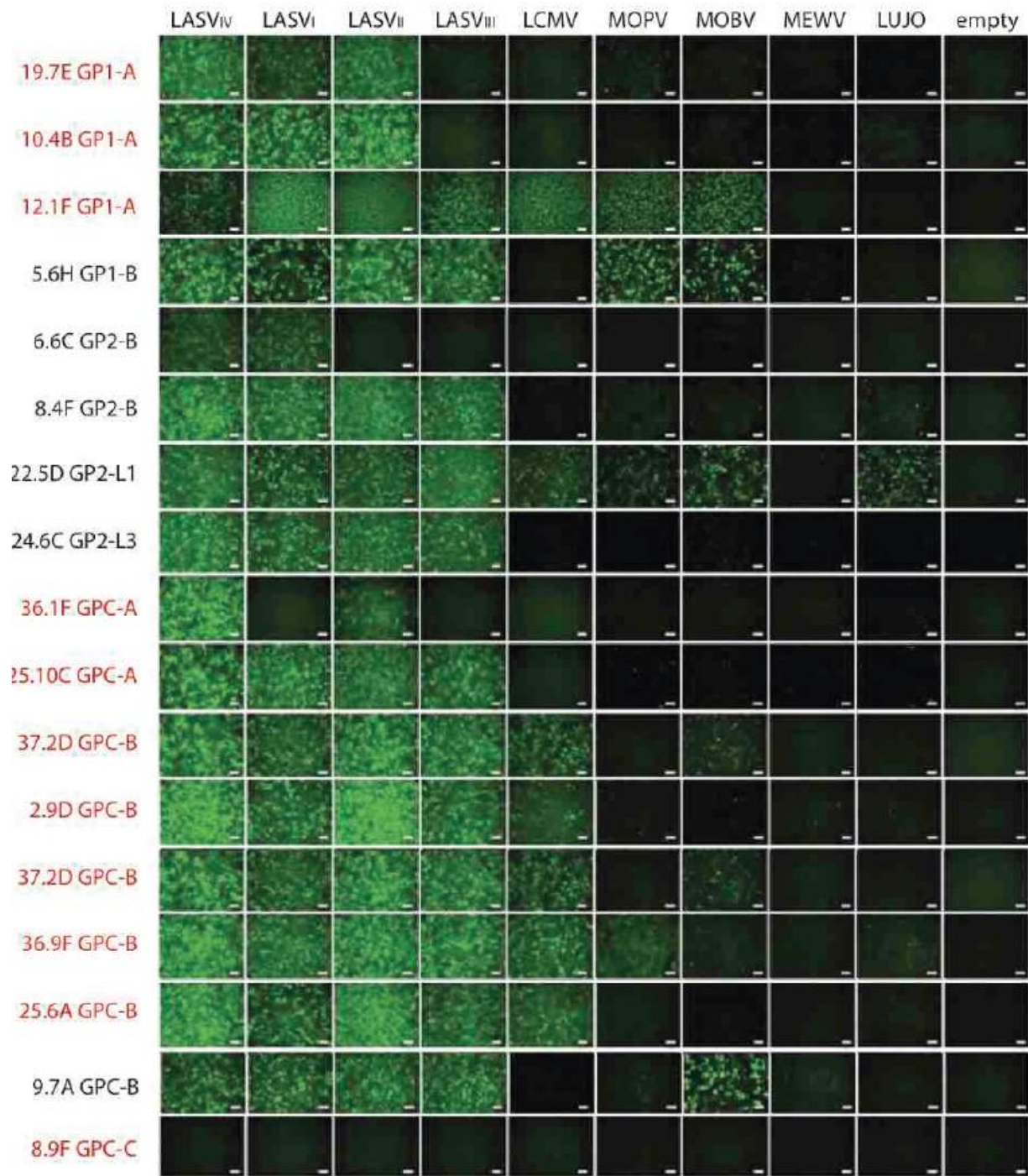

Supplementary Figure 7. Cross-reactivity of MABs isolated from survivors of LASV lineage IV infection with glycoproteins of other arenaviruses. Cross-reactivity of human MABs produced after natural infection with LASV lineage IV was investigated by immunofluorescence assays. Eukaryotic expression vectors encoding GPCs of Old World arenaviruses, including LASV lineages I - IV, LCMV, Mobala virus (MOBV), Mopeia virus (MOPV), Merino Walk virus (MEWV), and Lujo virus (LUJV) were transfected into HEK293T cells. Fixed cell monolayers were then incubated with LASV MABs and MAB binding was detected by indirect immunofluorescence. A selected subset of MABs is presented in Fig. 4a without results for MEWV. 8.9F was reactive with unfixed cells expressing LASV GPC (Fig. 1b), but was not reactive with cells expressing LASV GPC after the fixation conditions used in these experiments. Scale bar equals 100 μ m.

a

	LASV	LCMV	LUJO	MACV
10.4B GP1-A	+	+	-	-
19.7E GP1-A	+	-	-	-
12.1F GP1-A	+	+	-	-
3.3B GP1-B	+	-	-	-
2.4F GP1-B	+	-	-	-
21.5F GP1-B	+	±	-	-
5.0H GP1-B	+	-	-	-
18.5D GP1-B	+	-	-	-
39.3G GP1-B	+	±	-	-
6.6C GP2-B	+	-	-	-
13.9H GP2-B	+	+	-	-
37.2H GP2-B	+	+	+	+
6.9A GP2-B	+	+	+	-
3.0H GP2-B	+	+	-	-
14.10H GP2-B	+	-	-	-
8.4F GP2-B	+	-	-	-
22.5D GP2-L1	+	+	+	+
8.8B GP2-L1	+	+	+	+
13.4E GP2-L2	+	+	+	+
24.6C GP2-L3	+	±	-	-
25.10C GPC-A	+	-	-	-
36.1F GPC-A	+	-	-	-
37.2D GPC-B	+	+	-	-
36.9F GPC-B	+	+	-	-
18.5C GPC-B	+	+	-	-
37.7H GPC-B	+	+	-	-
37.2G GPC-B	+	+	-	-
2.9D GPC-B	+	+	-	-
25.6A GPC-B	+	+	-	-
8.11C GPC-B	+	+	-	-
9.7A GPC-B	+	+	-	-
8.9F GPC-C	-	-	-	-

c

	Del1<br>Δ261<br>-289	Del2<br>Δ261<br>-297	Del3<br>Δ261<br>-311	Del4<br>Δ261<br>-320	Del5<br>Δ261<br>-328	Del6<br>Δ261<br>-340	Del7.1<br>Δ261<br>-354	Del7.2<br>Δ261<br>-359	Del7.3<br>Δ261<br>-361	Del8<br>Δ261<br>-366	Del9<br>Δ261<br>-377
10.4B GP1-A	141	161	109	110	139	146	160	171	171	118	121
19.7E GP1-A	34	47	44	46	40	59	70	78	78	47	50
12.1F GP1-A	81	15	20	20	14	17	20	19	19	12	10
3.3B GP1-B	144	196	130	207	176	182	185	190	190	137	137
2.4F GP1-B	139	114	98	89	99	99	104	110	110	96	98
22.2G GP2-B	179	137	181	141	8	7	7	6	6	9	7
13.9H GP2-B	170	305	173	132	7	5	6	5	5	6	8
37.2H GP2-B	160	246	145	124	8	5	5	5	5	5	5
31.1H GP2-B	204	346	203	138	10	7	7	6	6	8	7
3.6H GP2-B	178	323	181	113	8	6	7	6	6	7	7
26.9C GP2-B	169	278	168	103	7	5	5	5	5	7	6
23.10A GP2-B	185	320	171	110	7	5	5	5	5	5	5
4.1F GP2-L1	115	141	4	3	4	3	3	2	2	3	3
8.8B GP2-L1	129	346	5	4	7	3	3	3	3	6	4
13.4E GP2-L2	116	133	109	109	114	118	113	123	123	100	3
1.1B GP2-L2	120	134	111	103	118	169	123	134	134	107	3
24.6C GP2-L3	150	209	112	107	145	113	112	119	119	123	125
25.10C GPC-A	98	3	2	3	3	2	2	3	3	2	2
36.1F GPC-A	108	5	3	4	4	3	3	4	4	3	3
8.11G GPC-A	45	6	8	4	6	7	3	6	6	6	3
37.2D GPC-B	56	12	21	12	24	8	16	15	15	6	7
9.8A GPC-B	53	14	21	25	33	18	53	43	43	3	3
36.9F GPC-B	85	41	50	63	67	46	84	90	90	3	2
18.5C GPC-B	100	53	51	73	61	42	82	88	88	3	2
NE13 GPC-B	83	29	34	46	49	26	60	53	53	2	2
37.7H GPC-B	85	34	36	46	50	26	83	53	53	3	3
37.2G GPC-B	NT	NT	42	60	NT	40	83	89	89	3	3
2.9D GPC-B	80	34	47	57	62	36	76	83	83	3	3
25.6A GPC-B	77	29	42	55	57	34	76	81	81	3	3
20.5G GPC-B	88	35	45	59	66	37	80	92	92	4	3
38.8B GPC-B	82	50	66	77	86	64	95	103	103	3	6
8.9F GPC-C	4	4	5	4	4	5	5	5	5	4	4

b

	GP1<br>N-term<br>Δ80<br>-75	fusion<br>loop<br>Δ280<br>-291	HR1<br>Δ311<br>-355	T loop<br>Δ365<br>-384	HR2<br>Δ400<br>-412
10.4B GP1-A	38	50	81	55	59
19.7E GP1-A	19	25	40	17	25
12.1F GP1-A	4	12	5	4	5
3.3B GP1-B	53	51	137	110	106
2.4F GP1-B	84	95	63	51	66
22.2G GP2-B	86	127	7	8	156
13.9H GP2-B	88	123	6	6	57
37.2H GP2-B	90	118	5	5	49
31.1H GP2-B	110	146	7	8	66
3.6H GP2-B	91	126	7	8	59
26.9C GP2-B	93	120	5	8	54
23.10A GP2-B	92	122	5	7	56
4.1F GP2-L1	96	98	4	113	108
8.8B GP2-L1	97	85	5	147	123
13.4E GP2-L2	101	100	101	4	111
1.1B GP2-L2	140	136	127	2	107
24.6C GP2-L3	100	99	106	179	4
25.10C GPC-A	2	8	2	3	2
36.1F GPC-A	4	8	3	5	4
8.11G GPC-A	6	4	6	7	4
37.2D GPC-B	8	13	9	0	7
9.8A GPC-B	6	19	24	3	2
36.9F GPC-B	2	39	56	2	2
18.5C GPC-B	3	39	57	2	2
NE13 GPC-B	2	25	40	2	2
37.7H GPC-B	3	26	35	3	3
37.2G GPC-B	2	38	66	3	2
2.9D GPC-B	2	33	41	2	3
25.6A GPC-B	2	32	43	2	2
20.5G GPC-B	2	27	42	3	3
38.8B GPC-B	NT	NT	NT	NT	NT
8.9F GPC-C	4	5	6	4	5

d

	63KGV	66YEL	69QTL	71ELN	75MET	77LMN	83PLS	90SHH	91HHY	93YIM	96VGN
10.4B GP1-A	113	137	140	85	152	100	141	142	82	22	61
19.7E GP1-A	63	71	82	45	188	53	71	172	62	15	39
12.1F GP1-A	23	7	22	5	116	4	7	3	4	4	9
3.3B GP1-B	202	217	217	182	200	201	214	219	163	73	163
2.4F GP1-B	91	98	95	66	99	78	104	95	93	17	57
22.2G GP2-B	179	247	241	172	176	114	144	145	82	41	88
13.9H GP2-B	173	238	211	165	169	112	146	154	80	39	92
37.2H GP2-B	172	220	206	159	163	114	144	145	82	41	88
31.1H GP2-B	230	306	284	217	175	150	186	190	73	42	119
3.6H GP2-B	232	305	299	227	217	153	198	203	104	49	130
26.9C GP2-B	202	252	240	187	178	126	165	166	94	47	105
23.10A GP2-B	200	250	238	182	187	121	159	162	84	40	102
4.1F GP2-L1	139	154	156	137	146	133	149	148	123	101	112
8.8B GP2-L1	135	134	141	133	133	133	143	138	123	124	123
13.4E GP2-L2	138	140	137	132	129	124	136	129	123	112	104
1.1B GP2-L2	198	206	208	189	182	181	198	181	164	167	139
24.6C GP2-L3	129	127	129	126	123	120	127	122	118	112	91
25.10C GPC-A	37	2	19	2	97	2	2	59	101	2	29
36.1F GPC-A	23	3	14	3	10	3	3	54	100	3	30
8.11G GPC-A	12	3	12	3	85	3	2	46	151	4	16
37.2D GPC-B	37	3	20	4	99	12	17	61	98	9	24
9.8A GPC-B	35	6	24	6	104	24	33	66	96	23	28
36.9F GPC-B	37	3	30	6	99	66	81	88	100	64	70
18.5C GPC-B	43	6	26	6	105	67	84	92	103	63	63
NE13 GPC-B	28	2	20	6	102	46	61	79	103	46	56
37.7H GPC-B	44	3	27	7	102	50	66	84	103	47	62
37.2G GPC-B	34	4	26	3	100	65	82	87	101	63	71
2.9D GPC-B	36	3	26	4	113	67	87	93	111	60	71
25.6A GPC-B	40	3	27	4	104	57	73	85	106	53	59
20.5G GPC-B	3	3	12	8	104	57	76	73	84	43	46
38.8B GPC-B	3	4	21	18	102	72	90	84	85	60	63
8.9F GPC-C	16	3	9	5	116	3	4	29	103	4	7

Supplementary Figure 8. Mutagenesis mapping of putative epitopes recognized by LASV MAbs by deletion and site-directed mutagenesis. Wild-type recombinant LASV GPC was engineered with deletions or site-directed mutations to map putative B cell epitopes on LASV glycoproteins. **Panel a:** GPC from the Old World arenaviruses LASV (Josiah strain, lineage IV), LCMV, Lujo and the New World arenavirus MACV were expressed and purified. Reactivity of the indicated MAbs was assessed by ELISA. A selected panel of MAbs is presented in Fig. 4b. 8.9F was not reactive with expressed and purified LASV glycoprotein. **Panel b:** ELISA reactivity of selected MAbs to constructs containing deletions of the N terminus of GP1 and structural features of GP2. A panel of selected MAbs is presented in Fig. 5c. Deletions that reduced binding to 10% or less of control or 11- 24% of control are highlighted. **Panel c:** ELISA reactivity of selected MAbs to constructs containing increasing larger deletions in GP2. A selected panel of MAbs is presented in Fig. 5d. **Panel d:** Binding of MAbs to GPC constructs in which the indicated sets of three amino acids in the amino terminus of GP1 were mutated to three alanines. A selected panel of MAbs is presented in Supplementary Fig. 6b.

Supplementary Table 1. LASV Glycoprotein MABs isolated from human survivors of Lassa fever

Group	huMAB	Neut-<br>ralizing	Epitope type	Putative<br>epitope<br>(amino acids)	Case	CDR<br>H1-3<br>lengths	HC<br>Mutation<br>%	CDR<br>L1-3<br>lengths	LC<br>Mutation<br>%	Comments
GP1-A	19.7E	+	conformational	111-117	G583	8.8.12	92.36	6.3.9	94.62	Loop between $\beta 4$ and $\alpha 1$
GP1-A	10.4B	+	conformational	111-117	G583	8.8.13	88.89	6.3.9	87.81	Neutralizing in PRNT assays only. Loop<br>between $\beta 4$ and $\alpha 1$
GP1-A	12.1F	+	conformational	111-117	G448	8.7.18	91.23	6.3.9	92.47	Loop between $\beta 4$ and $\alpha 1$, Conformation<br>sensitive, more complex epitope than other<br>GP1-A MABs, blocks 8.9F binding
GP1-B	21.6G	-	conformational	119-134	G355	8.8.17	93.4	9.3.9	98.26	$\alpha 1$ and $\alpha 2$
GP1-B	2.4F	-	conformational	119-134	G583	8.8.22	96.53	8.3.8	98.97	$\alpha 1$ and $\alpha 2$
GP1-B	5.11A	-	conformational	119-134	G583	8.8.19	95.14	6.3.11	92.83	$\alpha 1$ and $\alpha 2$
GP1-B	36.7D	-	conformational	119-134	G355	9.7.19	91.31	6.3.9	96.77	$\alpha 1$ and $\alpha 2$
GP1-B	19.5A	-	conformational	119-134	G583	10.7.14	92.44	6.3.9	94.62	$\alpha 1$ and $\alpha 2$
GP1-B	21.5F	-	conformational	119-134	G355	8.8.19	90.97	6.3.10	94.62	$\alpha 1$ and $\alpha 2$
GP1-B	3.3B	-	conformational	119-134	G502	8.8.25	64.93	tbd	tbd	$\alpha 1$ and $\alpha 2$
GP1-B	5.6H	-	conformational	119-134	G583	10.7.14	tbd	6.3.9	97.85	$\alpha 1$ and $\alpha 2$
GP1-B	18.5D	-	conformational	119-134	G617	10.7.22	98.28	6.3.11	97.85	$\alpha 1$ and $\alpha 2$
GP1-B	39.3G	-	conformational	119-134	G355	8.8.17	95.49	9.3.9	98.61	$\alpha 1$ and $\alpha 2$
GP1-B	9.9F	-	conformational	119-134	G673	10.7.15	90.03	6.3.9	91.76	$\alpha 1$ and $\alpha 2$
GP1-B	37.4E	-	conformational	119-134	G355	10.7.16	96.22	9.3.9	94.44	$\alpha 1$ and $\alpha 2$
GP2-A	7.1B	-	conformational	312-320	G634	8.8.18	96.88	9.3.10	97.22	HR1a
GP2-B	2.10G	-	conformational	328-358	G400	8.8.20	95.49	6.3.11	94.98	Two cysteines in CDR-H3. HR1
GP2-B	8.4F	-	conformational	328-358	G610	8.8.17	95.49	9.3.10	95.49	Two cysteines in CDR-H3. HR1
GP2-B	6.6C	-	conformational	328-358	LS011	8.8.22	94.10	7.3.8	90.78	Two cysteines in CDR-H3. HR1
GP2-B	37.9D	-	conformational	328-358	G482	8.8.23	98.21	6.3.10	98.21	Two cysteines in CDR-H3. HR1
GP2-B	37.2H	-	conformational	328-358	G355	8.8.19	92.71	12.3.9	93.27	HR1
GP2-B	6.7B	-	conformational	328-358	G583	8.8.19	96.53	9.3.10	97.22	HR1
GP2-B	6.9A	-	conformational	328-358	G551	8.8.17	94.79	6.3.9	96.06	HR1
GP2-B	20.4F	-	conformational	328-358	G551	8.8.17	93.4	6.3.9	94.27	HR1
GP2-B	13.9H	-	conformational	328-358	G551	8.8.16	95.49	6.3.8	97.49	HR1
GP2-B	3.6H	-	conformational	328-358	G355	8.8.17	92.01	9.3.11	97.37	HR1
GP2-B	26.9C	-	conformational	328-358	G355	8.8.15	90.62	6.3.8	94.27	HR1
GP2-B	31.1H	-	conformational	328-358	G482	8.7.11	95.09	6.3.8	94.27	HR1
GP2-B	23.10A	-	conformational	328-358	G482	8.8.15	96.53	6.3.10	97.85	HR1
GP2-B	22.2G	-	conformational	328-358	G482	8.7.20	91.23	9.3.11	94.79	HR1
GP2-B	14.10H	-	conformational	328-358	G482	8.8.13	97.57	8.3.11	97.59	HR1
GP2-L1	22.5D	-	linear	300-315	G355	8.8.17	95.49	11.3.9	96.94	Broadly cross reactive
GP2-L1	4.1F	-	linear	303-309	G355	8.8.14	92.71	7.3.9	93.26	Broadly cross reactive
GP2-L1	8.8B	-	linear	303-309	G400	8.8.13	94.44	8.3.11	96.22	Broadly cross reactive
GP2-L1	26.5E	-	linear	303-309	G355	8.8.11	91.67	12.3.9	96.30	Broadly cross reactive
GP2-L2	13.4E	-	linear	369-373	G482	8.8.12	90.28	9.3.12	96.18	Linear epitope: T-loop
GP2-L2	1.1B	-	linear	369-373	NG04	10.7.9	93.47	6.3.10	93.91	Linear epitope T-loop, Nigerian survivor
GP2-L3	24.6C	-	linear	401-415	G355	8.8.17	97.92	9.3.10	95.83	HR2
GPC-A	8.11G	+	conformational	62-68;<br>270-278	LS011	8.7.13	90.53	6.3.9	88.53	GP1 N term and fusion loop
GPC-A	25.10C	+	conformational	62-68;<br>270-278	G355	8.8.14	91.32	6.3.7	93.19	GP1 N term and fusion loop
GPC-A	36.1F	+	conformational	62-68;<br>270-278	G355	8.8.18	87.02	7.3.10	93.62	GP1 N term and fusion loop
GPC-B	37.7H	+	conformational	66-72;<br>364-371	G355	8.7.17	66.32	9.3.10	94.10	Similar HC to 25.6A.<br>GP1 N term and T-loop
GPC-B	25.6A	+	conformational	66-72;<br>364-371	G355	8.7.17	65.26	9.3.10	93.75	Similar HC to 37.7H<br>GP1 N term and T-loop
GPC-B	36.9F	+	conformational	66-72;<br>364-371	G355	8.8.16	91.32	12.3.9	63.64	Similar HC to 2.9D<br>GP1 N term and T-loop
GPC-B	2.9D	+	conformational	66-72;<br>364-371	G355	8.8.16	93.06	12.3.9	98.99	Similar HC to 36.9F<br>GP1 N term and T-loop
GPC-B	37.2G	+	conformational	66-72;<br>364-371	G355	8.8.16	93.75	7.3.10	96.45	GP1 N term and T-loop
GPC-B	18.5C	+	conformational	66-72;<br>364-371	LS011	8.8.15	88.89	7.3.10	92.55	GP1 N term and T-loop
GPC-B	9.8A	+	conformational	66-72;<br>364-371	NG05	8.8.17	95.83	6.3.9	96.42	Nigerian. GP1 N term and T-loop
GPC-B	37.2D	+	conformational	66-72;<br>364-371	G355	8.8.21	89.24	6.3.9	94.27	GP1 N term and T-loop
GPC-B	NE13	+	conformational	66-72;<br>364-371	NG05	8.8.15	96.53	7.3.8	94.68	Nigerian. GP1 N term and T-loop
GPC-B	9.7A	-	conformational	66-72;<br>364-371	G551	8.8.12	94.10	9.3.8	94.10	GP1 N term and T-loop
GPC-B	8.11C	-	conformational	66-72;<br>366-371	G551	8.8.12	93.75	9.3.8	93.75	GP1 N term and T-loop
GPC-B	10.5G	-	conformational	66-72;<br>364-371	G673	8.8.17	92.01	9.3.10	93.06	GP1 N term and T-loop
GPC-C	8.9F	+	conformational	tbd	LS011	9.7.31	92.01	9.3.10	94.10	Non-reactive in screening ELISA or with fixed<br>cells. Highly sensitive to conformation

SUPPLEMENTARY NOTE 1

The Viral Hemorrhagic Fever Consortium includes: Autoimmune Technologies, LLC: Dr. Kevin Simpson, Mike Charbonnet; Broad institute of MIT and Harvard University: Michael Butts, Chris Matranga, Kayla Barnes; Corgenix Medical Corporation: Douglass Simpson, Dan Simpson; The Scripps Institute: Dr. Michael B. A. Oldstone, Dr. Brian Sullivan; Kenema Government Hospital Lassa Fever Team includes: Jeneba Abu, Amadu Allieu, James Bangura, Baindu Dukullay, Mohamed Fomgbeh, Bockerie Kamara, Fatmata Kamara, Gabriel Kainesie, Marian Kallon, Tiangay Kallon, Kandeh Kargbo, Omaru Karbgo, James Koninga, Vandy Konneh, Victor Lungay, Ensa Massaquoi, Joseph Moseray, Lina Moses, Juma Musa, Willie Robert, Senesie Samai, Patrick Sannoh, John Sesay, Sheku Shaw, Mohamed Sow; Redeemer's University and Irrua Specialist Teaching Hospital Lassa fever Program: Donatus Adomeh, Odegua Adun, Jacqueline Agbukor, Micheal Airende, Dr. George O. Akpede, Chris Aire, Isreal Akhaine, Dr. Danny Asogun, John Ayepada, Tobin Ekaete, Solomon Ehikhametalor, Philomena Ehiane Eromon, Veritas Ifeh, Ganiyu Ighenegbale, Yemisi Olatayo Ighodalo, Ekene Muoebonam, Dr. Peter O. Okokhere, Thomas Olorok, Emmanuel Omomoh, Omowunmi Omoniwa, Jennifer Oyakhilome, Eghosa Uyigue; Tulane University: Sue Hutto, Crystal Gaither, Shirley Tubre', Trevor V. Gale, Andrew Hoffmann, Brandon Beddingfield, Lilia I. Melnik, Allyson M. Haislip; University of California at San Diego: Dr. Sheng Li; University of Texas Medical Branch: Chad E. Mire; Vybion, Inc.: Dr. Wallace Fish.

SUPPLEMENTARY REFERENCES

1. Hastie KM, *et al.* Crystal structure of the prefusion surface glycoprotein of the prototypic arenavirus LCMV. *accepted for publication*
2. York J, Nunberg JH. A novel zinc-binding domain is essential for formation of the functional Junin virus envelope glycoprotein complex. *J Virol* **81**, 13385-13391 (2007).
3. Cohen-Dvashi H, Cohen N, Israeli H, Diskin R. Molecular Mechanism for LAMP1 Recognition by Lassa Virus. *J Virol* **89**, 7584-7592 (2015).
4. Mahmutovic S, *et al.* Molecular Basis for Antibody-Mediated Neutralization of New World Hemorrhagic Fever Mammarenaviruses. *Cell Host & Microbe* **18**, 705-713 (2015).
5. Seiler P, Senn BM, Brundler MA, Zinkernagel RM, Hengartner H, Kalinke U. In vivo selection of neutralization-resistant virus variants but no evidence of B cell tolerance in lymphocytic choriomeningitis virus carrier mice expressing a transgenic virus-neutralizing antibody. *J Immunol* **162**, 4536-4541 (1999).
6. Weber EL, Buchmeier MJ. Fine mapping of a peptide sequence containing an antigenic site conserved among arenaviruses. *Virology* **164**, 30-38 (1988).
7. Igonet S, *et al.* X-ray structure of the arenavirus glycoprotein GP2 in its postfusion hairpin conformation. *Proc Natl Acad Sci U S A* **108**, 19967-19972 (2011).
